# Genomic and Epigenomic Responses to Chronic Stress Involve miRNA-Mediated Programming

**DOI:** 10.1371/journal.pone.0029441

**Published:** 2012-01-24

**Authors:** Olena Babenko, Andrey Golubov, Yaroslav Ilnytskyy, Igor Kovalchuk, Gerlinde A. Metz

**Affiliations:** 1 Department of Biological Sciences, University of Lethbridge, Lethbridge, Alberta, Canada; 2 Canadian Centre for Behavioural Neuroscience, University of Lethbridge, Lethbridge, Alberta, Canada; 3 Hotchkiss Brain Institute, University of Calgary, Calgary, Alberta, Canada; Kyushu Institute of Technology, Japan

## Abstract

Stress represents a critical influence on motor system function and has been shown to impair movement performance. We hypothesized that stress-induced motor impairments are due to brain-specific changes in miRNA and protein-encoding gene expression. Here we show a causal link between stress-induced motor impairment and associated genetic and epigenetic responses in relevant central motor areas in a rat model. Exposure to two weeks of mild restraint stress altered the expression of 39 genes and nine miRNAs in the cerebellum. In line with persistent behavioural impairments, some changes in gene and miRNA expression were resistant to recovery from stress. Interestingly, stress up-regulated the expression of Adipoq and prolactin receptor mRNAs in the cerebellum. Stress also altered the expression of Prlr, miR-186, and miR-709 in hippocampus and prefrontal cortex. In addition, our findings demonstrate that miR-186 targets the gene Eps15. Furthermore, we found an age-dependent increase in EphrinB3 and GabaA4 receptors. These data show that even mild stress results in substantial genomic and epigenomic changes involving miRNA expression and associated gene targets in the motor system. These findings suggest a central role of miRNA-regulated gene expression in the stress response and in associated neurological function.

## Introduction

Stress affects the function of most organs including the brain. Psychological challenges are among the most powerful stimuli to induce a cascade of complex neuroendocrine and autonomic changes [1]. Since 1914, when Walter Bradford Cannon first described the psychophysiology of the stress [2], abundance of data suggested that stress can induce lasting molecular and physiological changes in the brain and its output, behaviour. The brain represents a central regulator which controls the behavioural and physiological responses to stressful events [3]. In a chronic condition, these physiological responses have the potential to facilitate the onset and progression of disease.

Variability in the stress response and susceptibility to disease is influenced by the genetic and epigenetic background of each individual [4]. Epigenetic components, which regulate gene expression, include DNA methylation, histone modification, chromosome remodeling, and expression of small non-coding RNAs such as microRNA (miRNA). The understanding of the interaction between genetic and epigenetic components in the brain under a stressful condition can provide an insight into pathogenic processes that contribute to neurological diseases. For instance, miRNAs may be a contributing factor to aging-related neurodegenerative diseases [5], [6], [7]. It was shown that substantial loss of mature miRNAs in the cerebellum of Dicer knock-out mice causes progressive neurodegeneration [8]. At the same time exposure to stress can cause changes in epigenetic machinery. For example, maternal care alters epigenetic programming and can determine the offspring's adult stress response [9].

Our previous data suggest that 20 minutes of chronic mild psychological stress, induced by restraint, causes lasting impairments in skilled movement and balance in rats [10], [11]. Considering that motor impairments in male rats persist even after the cessation of the stressor [11], it is possible that epigenetic mechanisms may be involved to permanently alter movement performance via genomic changes in motor areas. The largest and one of the most important motor regions is the cerebellum, which contributes to the learning and coordination of skilled movements [12]. It is likely that stress-induced motor impairments are related to altered processing by the cerebellum. We hypothesized that impaired motor control by stress is related to changes in miRNA and protein-encoding gene expression. The results show that mild chronic psychological stress changes cerebellar miRNA and mRNA expression. We confirmed the expression of several mRNA and miRNAs and demonstrate that miR-186 targets Eps15. The expression of some genes and miRNA expression was also changed in hippocampus and prefrontal cortex. Thus, the present observations demonstrate that even mild stress results in substantial changes in the expression of mRNA and miRNA in the brain.

## Results

### Body weight and corticosterone levels

**Figure 1:**
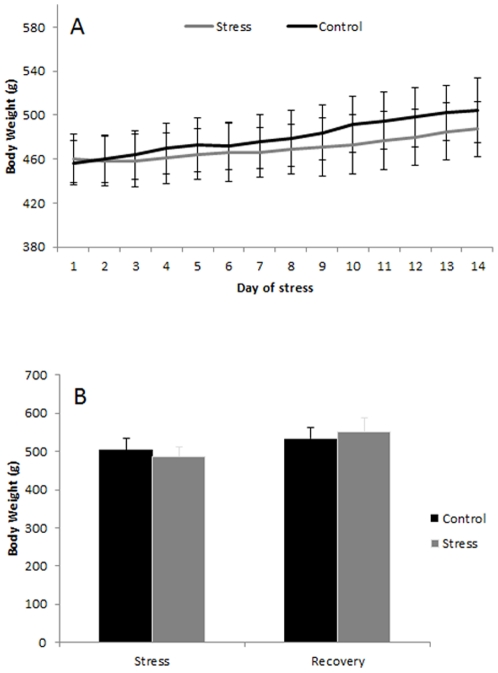
A: Body weight growth curves. The time course of body weight gain in animals undergoing two weeks of stress (gray) and non-stress control animals (black). Note that stress moderately diminished the average rate of weight gain (mean ± SD). B: Body weight (means ± SD) after two weeks of stress (“Stress”) and after two weeks of stress followed by two weeks of recovery (“Recovery”).

Body weight growth curves were not different between the control and the stress groups (Fig. 1). The mean weight of animals in the stress group (487.3±25.2 g) was 3.5% lower than that of the control group (504.5±29.2 g) (Fig. 1).

**Figure 2:**
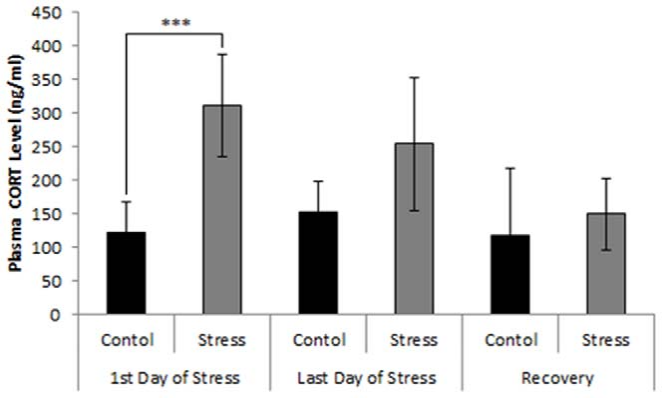
Concentration of plasma corticosterone (means ± SD; ng/ml) in control and stress animals as measured on the first day of stress, last day of stress and after two weeks of recovery from stress (“Recovery”). Asterisks represent statistical significance (*** p<0.001).

Analysis of the concentration of plasma corticosterone in control and stress animals showed significant differences (p<0.001) on the first day of stress (Fig. 2). On the last day of stress, stress animals had lower levels of corticosterone as compared to the first day of stress, indicating habituation to the stress procedure. Moreover, stress animals showed a decline in corticosterone levels after recovery from stress compared to the first day of stress. No difference between stress and control animals was found.

### Skilled reaching success

**Figure 3:**
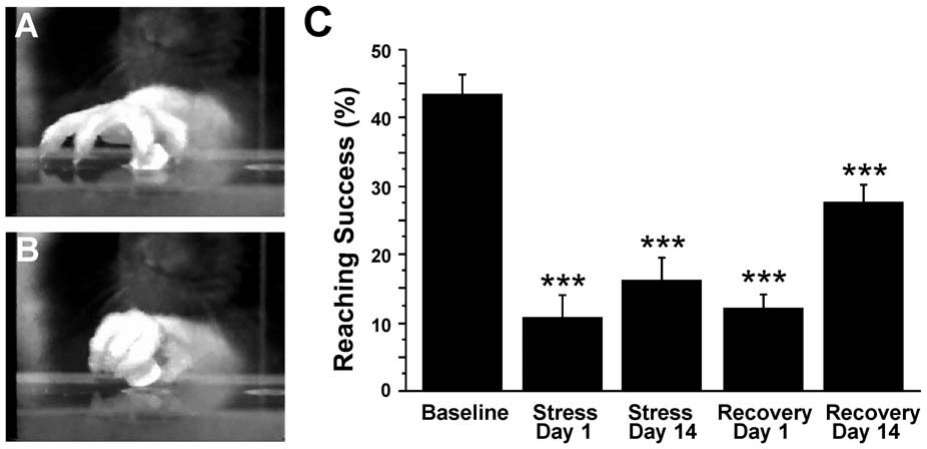
Skilled reaching performance in male rats. A, B: Series of photographs illustrating forelimb and digit movements of a rat grasping a food pellet. C: skilled reaching success in rats before (baseline), during and after two weeks of stress (mean ± SEM). Note that stress diminished skilled reaching performance after acute (day 1) and chronic (day 14) of daily stress treatment. Reaching success did not return to baseline levels within 14 days of recovery. Asterisks represent statistical significance (*** p<0.001).

There was a significant main effect of Group (F_4,28_ = 23.51, p<0.0001). Compared to baseline, stress reduced reaching success on the first day (day 1; t = 6.88, p<0.001) and last day of stress treatment (day 14; t = 9.02, p<0.0001) (Fig. 3). On the first day of recovery, rats still showed significantly reduced reaching success (t = 7.97, p<0.0001). Reaching success did not recover to baseline levels by day 14 of recovery from stress (t = 5.69, p<0.001). Compared to day 1 of the recovery period, however, performance improved by day 14 of recovery (t = 7.17, p<0.001).

### mRNA microarray analysis

mRNA expression pattern was analyzed in the following experimental groups: 2 weeks of daily restraint stress (2WSTRESS, n = 3), 2 weeks controls (2WCONTROL, n = 3), 2 weeks of daily restraint stress + 2 weeks of recovery from stress (4WSTRESS, n = 3), 4 weeks controls (4WCONTROL, n = 3).

**Figure 4:**
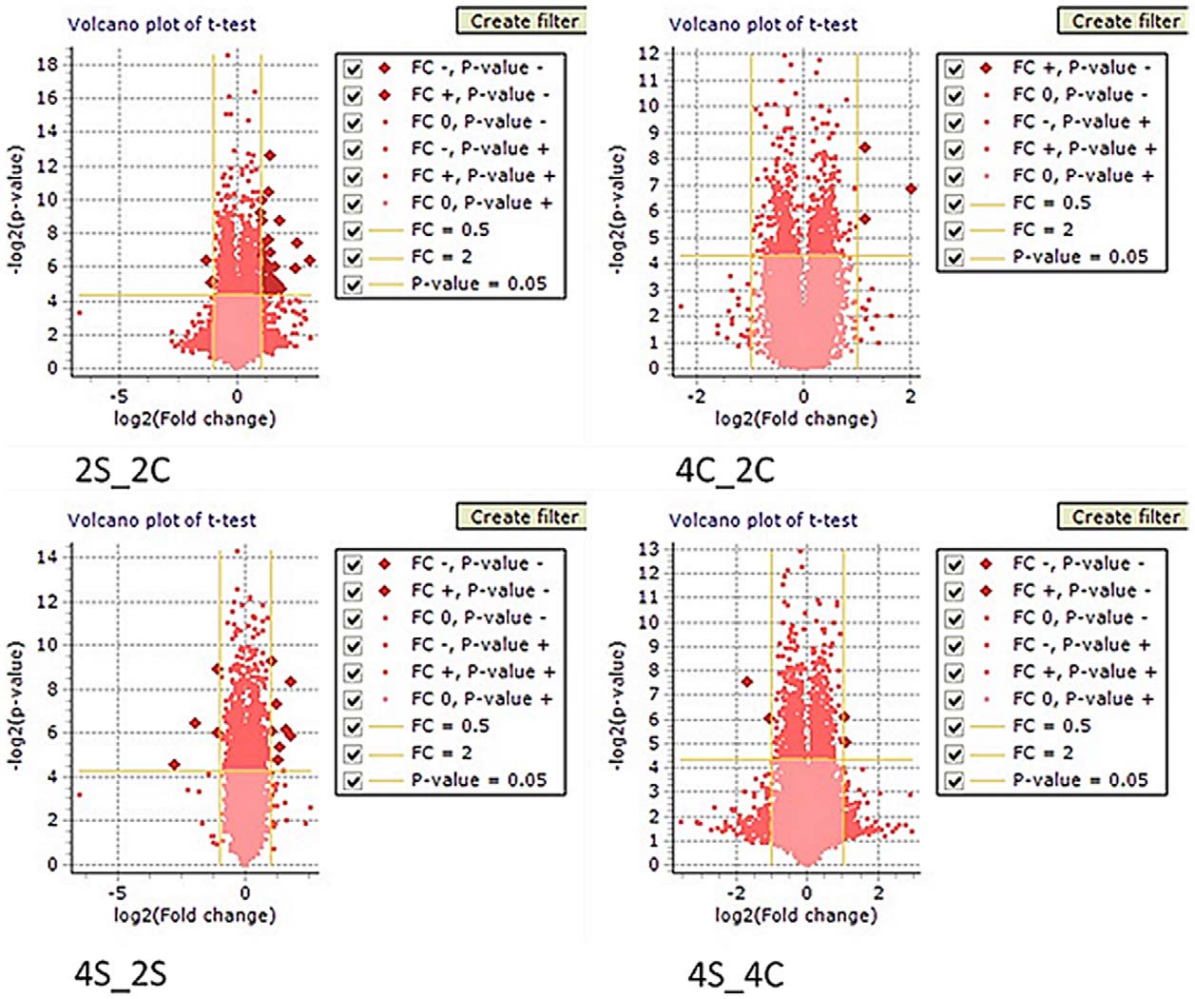
mRNA microarray expression analysis in animals after two weeks of stress (2WSTRESS), recovery from stress (4WSTRESS) and appropriate controls for each time point (2WCONTROL, 4WCONTROL). Genes with a 2-fold difference and a p-value of p<0.05 are shown in logarithmic scale (log_2_). Genes that were changed are represented as dark-red diamonds, in top-left (down-regulated) and top-right (up-regulated) parts of each figure. 2S_2C – groups of 2WSTRESS vs. 2WCONTROL, 4C_2C – 4WCONTROL vs. 2WCONROL, 4S_2S – 4WSTRESS vs. 2WSTRESS, 4S_2C – 4WSTRESS vs. 2WCONTROL.

Microarray data analysis showed that after two weeks of stress (2WSTRESS) 39 genes changed significantly (p<0.05; 2-fold difference) compared to respective controls (2WCONTROL): 36 were up-regulated, while three genes were down-regulated (see Table 1, Fig. 4). To exclude the possibility that these changes were due to changes in control values, 2WCONTROL vs. 4WCONTROL data were plotted, and only three genes remained changed, all being up-regulated. The changes observed upon comparison of RNA profile in the 2WSTRESS group of animals vs. 2WCONTROL of animals were nearly completely eliminated after two weeks of recovery after stress. A comparison of the expression in the 4WSTRESS group with the expression in the 4WCONTOL group showed just four genes were altered and two of them were down-regulated. All of these 4 genes were different from the group of 39 genes. In order to find some of these genes still changed in the 4WSTRESS group less stringent conditions were applied. Therefore, 4WSTRESS vs. 4WCONTROL groups were plotted using 1.3-fold change and a p-value<0.1 as cut-off. The generated list included 803 genes (data not shown) with 7 of them being the genes found in 2WSTRESS vs. 2WCONTROL comparison (Table 2).

**Table 1:** List of genes changed after two weeks of restraint treatment (2WSTRESS vs. 2WCONTROL).

Gene ID	log2 (Fold change)	P-value
TC2N	1.16	0.017
STRA6	1.32	0.001
CLCA3_PREDICTED	−1.31	0.012
NUDT7_PREDICTED	1.05	0.047
LOC497720	2.59	0.006
LOC689246	1.04	0.044
RGD1563952_PREDICTED	1.06	0.006
PI15_PREDICTED	1.40	0.000
CITED1	1.34	0.005
LOC500853	1.11	0.006
GDF15	1.50	0.025
CLDN3	1.52	0.032
MGC114520	1.73	0.031
ADIPOQ	1.04	0.002
RGD1562658_PREDICTED	1.16	0.027
LOC502201	1.04	0.009
ITGB6	1.06	0.002
OSMR	1.10	0.001
FOLR1	1.91	0.038
LOC361399	1.21	0.013
LOC501293	1.45	0.047
CLDN2_PREDICTED	2.52	0.016
LOC497820	−1.12	0.030
PRLR	1.34	0.018
LOC501363	1.12	0.014
MSX1	1.64	0.016
CDH3	1.01	0.014
TCF21	1.14	0.041
RGD1564074_PREDICTED	3.11	0.012
LOC317599	1.81	0.002
CAPN12_PREDICTED	−1.00	0.028
CORIN	1.17	0.035
LOC501482	1.03	0.028
CRB3	1.13	0.039
IQCG	1.04	0.009
SLC26A7_PREDICTED	1.19	0.036
LOC497864	1.28	0.014
OTC	1.59	0.040
RGD1563000_PREDICTED	1.41	0.009

**Table 2:** Comparison of gene expression levels between groups undergoing 2 weeks of stress (2WS) and controls (2WC) and recovery from stress (4WS) and controls (4WC).

Target ID	2WS vs. 2WC		4WS vs. 4WC	
	log2(Fold change)	P-value	log2(Fold change)	P-value
CRB3	1.132	0.039	0.828	0.088
NUDT7_PREDICTED	1.048	0.047	0.872	0.094
ADIPOQ	1.044	0.002	0.579	0.006
CDH3	1.008	0.014	0.682	0.086
OSMR	1.101	0.001	0.883	0.088
CORIN	1.172	0.035	0.746	0.058
LOC501482	1.027	0.028	0.497	0.045

### Semiquantitative RT-PCR confirmed the expression of *Prlr* and *Adipoq* genes

**Figure 5:**
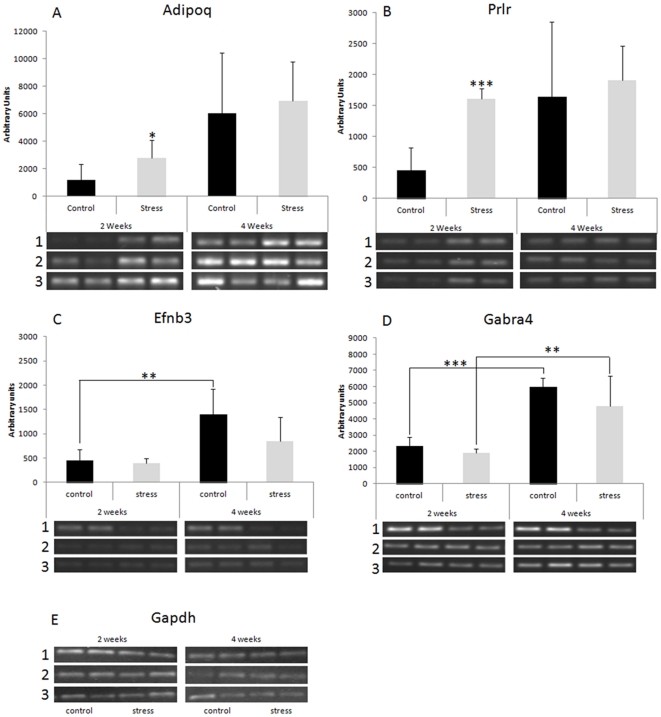
sq-RT-PCR analysis. A: prolactin receptor (*Prlr*) gene; B: *Adipoq* gene. C: ephrin B3 receptor (*Efnb3*) gene; D: GABA (A) receptor 4 (*Gabra4*) gene. E: GAPDH. Data are represented as an average of three animals per group. Asterisks represent statistical significance (* p<0.05; ** p<0.01; *** p<0.001). Error bars represent standard deviation of the mean. Photographs below bars represent corresponding PCR fragments in duplicates for each animal for three animals per group.

To confirm changes in mRNA expression level we chose two related to stress genes from the list of genes that changed after two weeks of stress (Table 1): prolactin receptor (*Prlr*) and adiponectin, C1Q and collagen domain containing gene (*Adipoq*). Gamma-aminobutyric acid (GABA) A receptor, alpha 4 gene (*Gabra4*) and ephrin B3 gene (*Efnb3*) were used as controls. sq-RT-PCR was performed in duplicates using the same RNA samples that were used for microarray analysis. Sq-RT-PCR data analysis confirmed a 2-fold difference between the stressed and non-stressed groups for both, *Adipoq* and *Prlr* genes (Fig. 5A, B). In contrast, no significant differences in expression of *Gabra4* and *Efnb3* were observed (Fig. 5C, D). Interestingly, there were significant age-dependent changes in the expression level of *Gabra4* and *Efnb3* genes. 4WSTRESS and 4WSTRESS samples showed higher expression than 2WSTRESS and 2WCONTROL (see Fig. 5C, D).

### Quantitative RT-PCR showed changes in *Prlr* expression in prefrontal cortex and hippocampus

**Figure 6:**
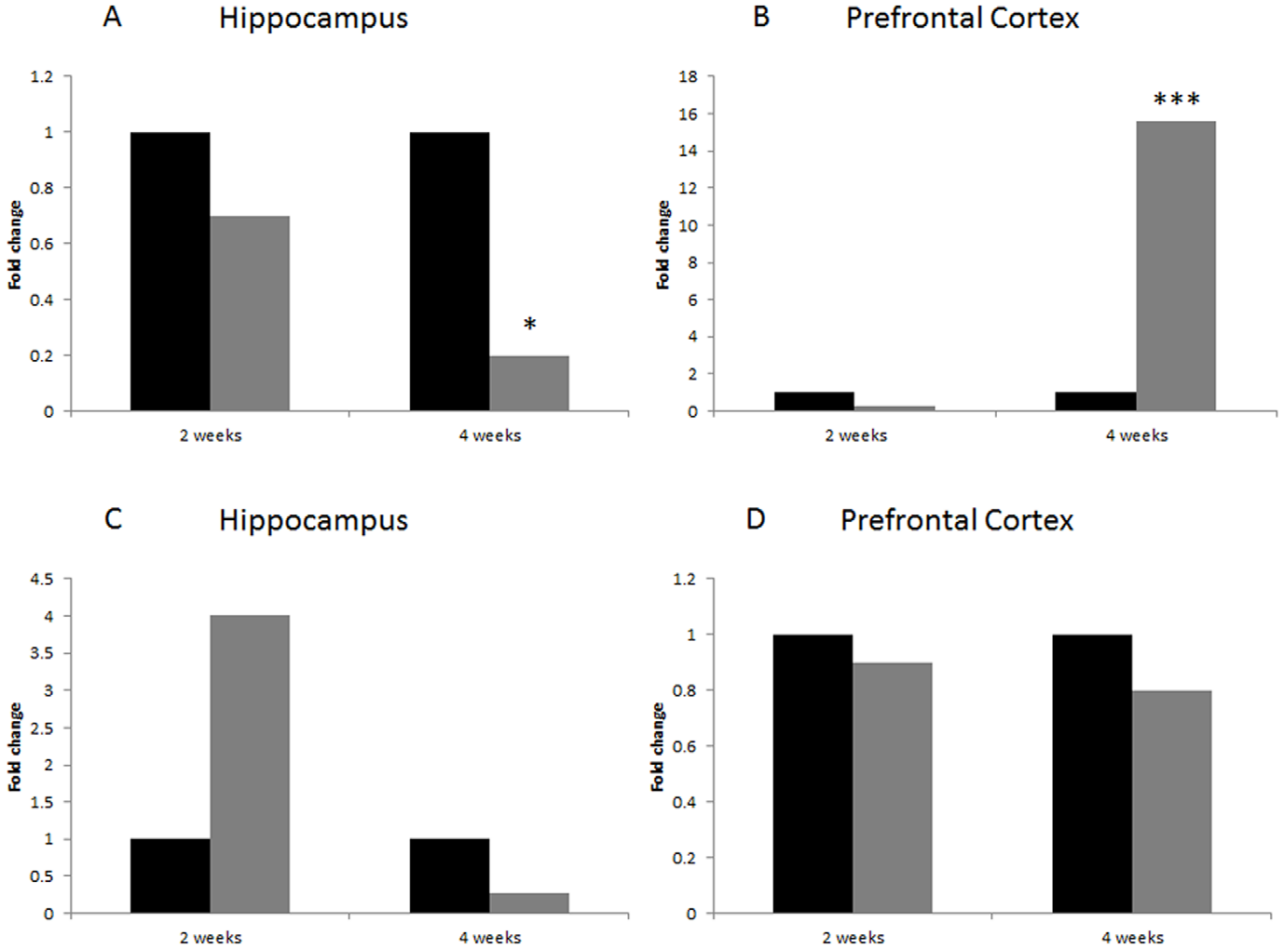
qRT-PCR analysis of *Prlr* (A, B) and *Adipoq* (C, D) expression level. Data are represented as a normalized relative fold change to control. Asterisks represent statistical significance (* p<0.01; *** p<0.001). Control animals are represented in black, stressed animals in grey. For more details see (Tables S3, S4, S5, S6). It should be noted that the level of Adipoq expression in hippocampus is very low (C(t) values >40 and beyond detection), which results in a high fold change that is not significant.

The expression level of *Prlr* and *Adipoq* genes was assessed in hippocampus and prefrontal cortex, brain regions important for the regulation of the stress response. There were no significant changes in *Prlr* expression in the prefrontal cortex or hippocampus after two weeks of stress (Fig. 6A, B). Interestingly, after two weeks of recovery from stress *Prlr* expression in the prefrontal cortex was up-regulated while it was down-regulated in the hippocampus (Fig. 6A, B). There were no significant changes in *Adipoq* expression in hippocampus or prefrontal cortex at either two or four weeks after stress (Fig. 6C, D). For more details see (Tables S1, S2, S3, S4, Figs. S1, S2). It should be noted, however, that the level of Adipoq expression in hippocampus is very low (C(t) values>40 and beyond detection), which results in a high fold change that is not significant. When dealing with very low copy numbers, the distribution of the template is not expected to be normal, instead, it follows a Poisson distribution. Thus, a large number of replicates are necessary in order to provide statistical significance (Application note, www.appliedbiosystems.com).

### Analysis of gene lists using DAVID Bioinformatics Resources

**Figure 7:**
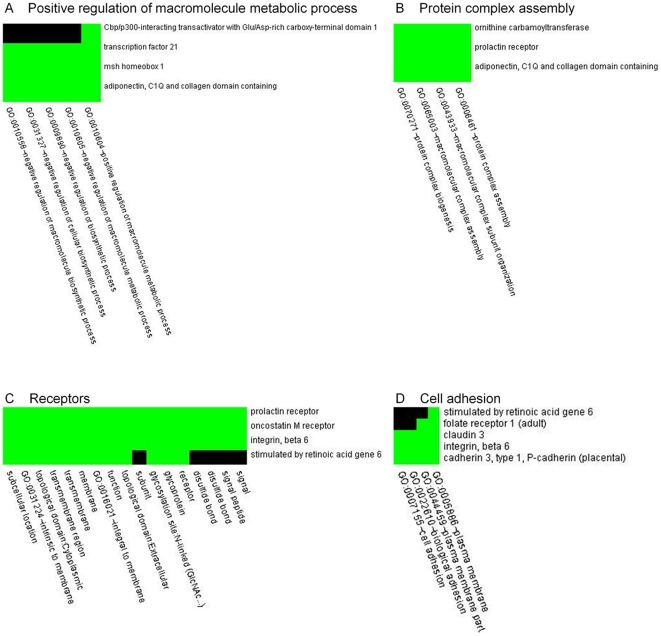
Functional annotation clustering. Green - corresponding gene-term association positively reported; black - corresponding gene-term association not reported yet. A: cluster of four genes: *Tcf21*, *Msx1*, *Adipoq*, *Cited*, which were grouped based on their involvement in positive regulation of the macromolecule metabolic process. B: cluster of three genes: *Prlr*, *Otc*, *Adipoq*, which were grouped based on their involvement in protein complex assembly. C: cluster of four receptors: *Prlr*, *Osmr*, *Itgb6*, *Ssta6*. D: cluster of three genes: *Cldn3*, *Itgb6*, *Cdh3*, which were grouped based on their involvement in cell adhesion.

To describe the possible role of the changes in regulation of 39 aforementioned genes, these genes were analysed using the Database for Annotation, Visualization and Integrated Discovery (DAVID) [13]. Predicted open reading frames (ORFs) were excluded from analysis by DAVID. The remaining 20 genes were grouped into four functional clusters: positive regulation of macromolecule metabolic process (genes: *Tcf21, Msx1, Adipoq, Cited1*), protein complex assembly (genes: *Prlr, Otc, Adipoq*), cell adhesion (genes: *Cldn3, Itgb6, Cdh3*), receptor (genes: *Prlr, Osmr, Itgb6, Ssta6*) (see Table S5 and Fig. 7).

### miRNA microarray analysis

**Figure 8:**
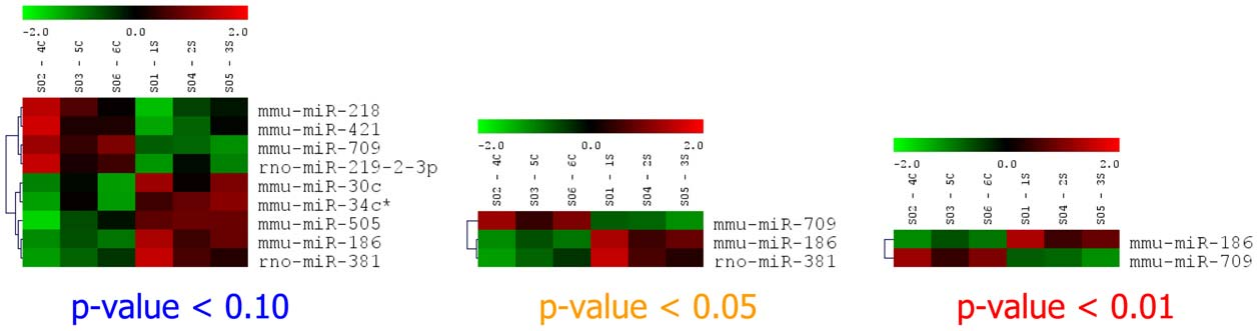
Analysis of miRNA expression in the rat cerebellum after two weeks of stress compared to controls. The microarray heatmap demonstrates the log_2_ ratio of miRNA signal difference between control and stress samples. Up-regulated miRNAs are shown in red, while down-regulated miRNAs are shown in green. The first three columns on each figure represent the level of expression in control animals, while the three last columns represent the level of corresponding miRNA expression in stress animals.

miRNA microarray analysis was performed using a µParaflo® Biochip, containing 832 mature miRNA sequences. The following samples were used for analysis: 2 weeks of daily restraint stress (2WSTRESS, n = 3) and 2 weeks controls (2WCONTROL, n = 3). First, data with a p-value<0.1 were analyzed. We found that nine miRNAs were changed in stressed animals in comparison to controls (see Fig. 8). Three miRNAs changed significantly with a p-value<0.05 in comparison to controls. Two miRNAs, miR-186 and miR-381, were up-regulated, while miR-709 was down-regulated. For further analysis only miRNAs with the lowest p-value (p<0.01) were used, which included miR-186 (log_2_ ratio of 0.43) and miR-709 (log_2_ ratio of −0.66).

### qRT-PCR confirmed the expression of miR-186 and miR-709

**Figure 9:**
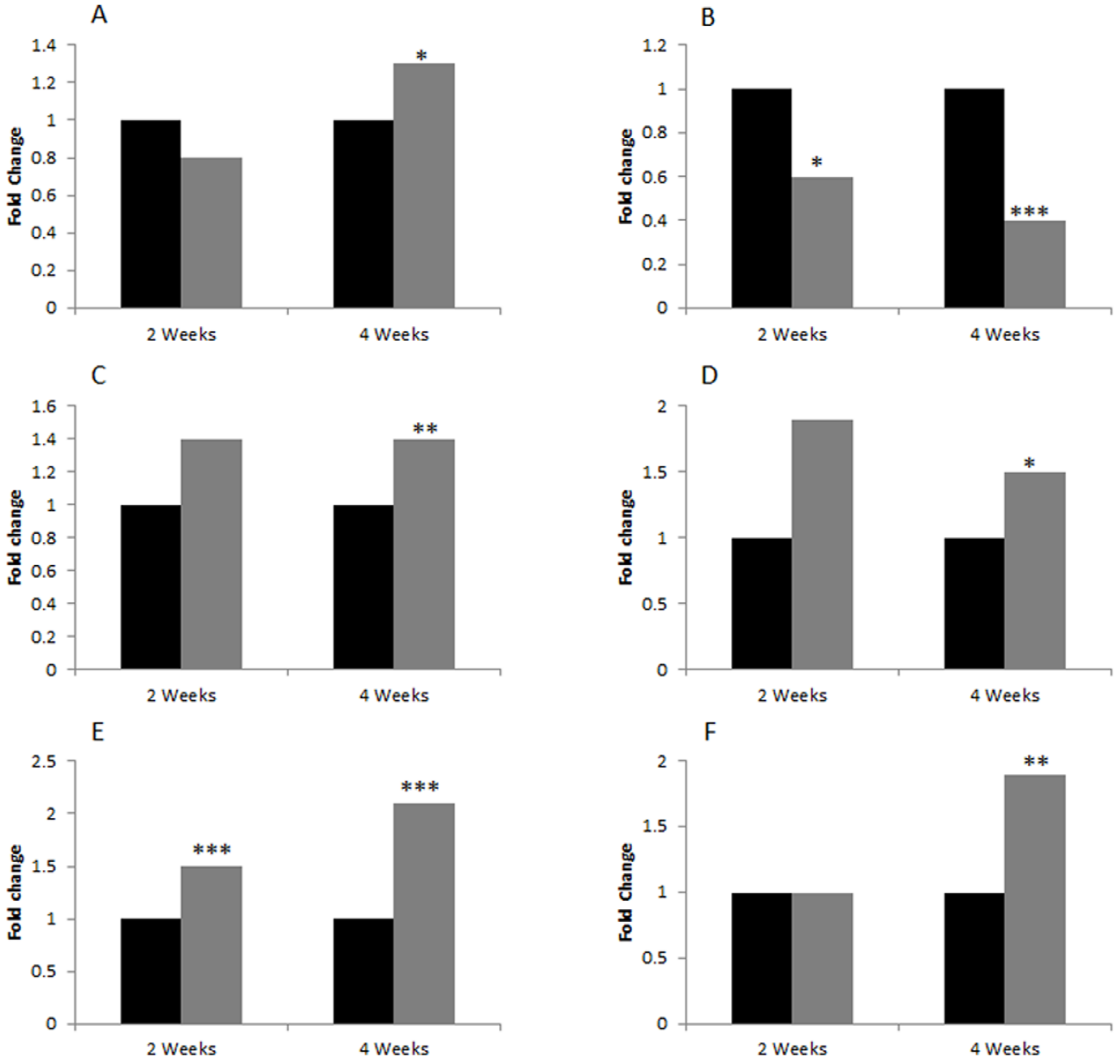
qRT-PCR analysis of miR-186 (A,C,E) and miR-709 (B,D,F) expression level in cerebellum (A,B), hippocampus (C,D), and prefrontal cortex (E,F). Data are represented as a normalized relative fold change to control. Asterisks represent statistical significance (*p<0.1; **p<0.05; ***p<0.001). Control animals are represented in black, stressed animals in grey. For more details see (Tables S7, S8, S9, S10, S11, S12).

We performed qRT-PCR analysis to confirm changes in miR-186 and miR-709 expression after two weeks of stress, and to investigate their expression after four weeks. The microarray results were confirmed for miR-709, which was down-regulated after two weeks of stress (2WSTRESS vs. 2WCONTROL) and demonstrated that this pattern of expression persists after two weeks of recovery from stress (4WSTRESS vs. 4WCONTROL). Interestingly, miR-709 showed different expression patterns in different brain regions (Fig. 9B, D, and F).

We did not observe any significant changes for miR-186 in stress and control animals after two weeks of stress, however, miRNA-186 was significantly up-regulated in cerebellum after two weeks of recovery from stress (Fig. 9A). miR-186 showed similar expression patterns in hippocampus and prefrontal cortex (Fig. 9C and E). For more details see (Tables S6, S7, S8, S9, S10, S11, Figs. S3, S4).

### Analysis of predicted miRNA targets

Currently there are no confirmed targets for miR-186 and miR-709 in the brain. Computational analysis of predicted targets for miR-186 revealed 365 putative miRNA targets, with a total of 398 conserved sites and 271 poorly conserved sites, with the score from −1.31 (most favorable) to 0.00 (data are not shown). We examined the first 50 genes with the most favorable score (<−0.59). Among those we chose five targets which may be important in the brain: *Gabra4* (score: −1.31), *Creb3* (−1.07), *Eps15* (−0.93), *A2bp1* (−0.65), and *Map3k2* (−0.81). We were able to clone the wild and mutated binding site only of *Eps15* into the plasmid used for Luciferase Reporter Assay, and thus we performed the analysis for *Eps15* only (see Fig. S2). The 3′UTR of the *Eps15* gene (NM_001009424) contains one binding site with a poorly conserved sequence for rno-miR-186 with one mismatch. The seed sequence is located 422–429 bp downstream of stop codon of *Eps15* CDS. To confirm that *Eps15* is indeed targeted by miR-186 we carried out a luciferase reporter assay.

**Figure 10:**
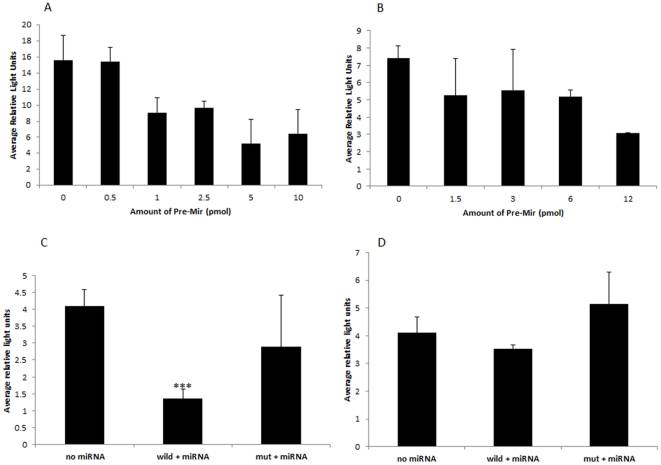
A: Dose-dependent inhibition of *Eps15* expression in the Luciferase Assay after transfection of HEK-293 cells with miR-186. B: Dose-dependent inhibition of the *Nab1* gene in the Luciferase Assay after transfection of MCF7 cells with miR-709. C: Luciferase Assay with pFN4 (3′UTR *Eps15*) and miR-186. The first bar demonstrates relative level of luciferase activity after transfection of MCF-7 cells with pFN4 only. Second bar: pFN4 + miR-186. Third bar: pFN4 mut + miR-186. Asterisks represent statistical significance (p<0.001). D: Luciferase Assay with pFN7 (3′UTR *Nab1*) and miR-709. The first bar demonstrates relative level of luciferase activity after transfection of MCF-7 cells with pFN7 only. Second bar: pFN7 + miR-709. Third bar: pFN7 mut + miR-709. Bars represent the normalized average of relative luciferase units.

Analysis of Luciferase Assay data showed that the luciferase activity was inhibited by miR-186 after co-transfection of mammalian cells with the construct carrying *Eps-15* 3′UTR in a dose-dependent manner (Fig. 10A). Co-transfection of mammalian cells with negative control (unrelated miRNA) revealed no changes in luciferase activity (Fig. 10C).

A similar approach was used to assess the putative target of miR-709 (3′UTR of *Nab1* gene). Computational analysis of predicted targets revealed 331 putative miRNA targets, with a total of 347 conserved sites and 193 poorly conserved, with the score from −0.81 (most favorable) to 0.00. We examined the first 50 genes that obtained the most favorable score (<−0.42). Among those we chose four targets which were reported of significant relevance to brain function: *Creb5* (−0.56), *Efnb3* (−0.6), *Nav1* (−0.49), and *Nab1* (−0.43). We were not able to clone wild and mutated seed sequences of *Efnb3*, *Nav1* and the mutated seed sequence of *Creb5*. Further work continued with *Nab1* only. The 3′UTR of *Nab1* gene (NM_022856) contains one binding site with a poorly conserved sequence for rno-miR-709. The binding site is located 84–91 bp downstream of stop codon of *Nab1* CDS. Analysis of the Luciferase Assay data revealed a tendency to decrease in expression of luciferase in the case of normal or mutated binding site, however, it was not significant (Fig. 10B, D).

## Discussion

The present study revealed that expression of 39 genes and nine miRNAs was changed after two weeks of restraint treatment. Furthermore, we verified one putative target for one of the changed miRNAs and expression of two selected genes. These data suggest that even a very mild stressor can cause molecular changes in the brain, which might play an important role in the onset and progression of neurological diseases.

### Physiological and behavioural changes

Chronic restraint stress in the present study caused elevated plasma corticosterone (CORT) levels on the first day of stress, which is in line with previously reported data [14], [15], [16]. Furthermore, we observed that animals show habituation to stress by 14 day of daily restraint treatment. The lack of chronic elevation is in accordance with the notion that rats may habituate to stress across subsequent exposures. For example, Magarinos and colleagues reported that daily chronic restraint stress causes a significant habituation by day 21 in the corticosterone response [17].

The present experiments also revealed no differences in body weight gain after two weeks of stress in the experimental group. There also were no differences between stress and control animals after two weeks of recovery from stress. These results are consistent with previous results showing an absence of body weight gain after two weeks of restraint [11]. Moreover, Jadavji and Metz (2008) showed corresponding behavioural changes in motor performance, which were confirmed by the present study. Interestingly, while females readily recovered from stress-induced impairment during a post-stress recovery period of two weeks, males did not recover [11]. We hypothesized that the long-lasting disturbances in motor performance in male rats might be caused by epigenetically regulated changes in gene expression in the cerebellum. Indeed the present results revealed that chronic stress causes changes in the gene and miRNA expression patterns in the brain.

### Chronic restraint stress causes changes in mRNA expression in cerebellum

Our results revealed that chronic restraint stress causes changes in gene expression in the cerebellum. After two weeks of stress we observed up-regulation in 36 genes, while three genes were down-regulated: *Clca3* predicted, LOC497820, *Capn12* predicted. Among the total number of changed genes 19 belong to the category of predicted genes. For further analysis using the Database for Annotation, Visualization and Integrated Discovery (DAVID) we considered only the remaining 20 genes with known function. The following will discuss some of the most prominent mRNA changes in detail.

Functional annotation clustering by DAVID revealed that changed genes can be grouped into four clusters: regulation of metabolic process, cell adhesion, protein complex assembly, and receptors. Surprisingly, all of them directly or indirectly relate to metabolism and signal transduction. Also some of these genes, such as *Tcf21* and *Osmr* were shown to be regulated by methylation and involved in carcinogenesis [18], [19]. One could speculate that the set of these four clusters could indicate a link between mild stress exposure and its possible outcomes, such as metabolic diseases (obesity, diabetes), cardiovascular diseases and anxiety. Anxiety may be an indirect regulator of motor skill performance [20], while altered signal transduction in the cerebellum might be directly related to stress-induced motor impairment and compensation of these deficits.

### Stress up-regulates the expression of Adipoq

Our results showed a two-fold up-regulation in the expression of *Adipoq*. *Adipoq* is a gene that encodes the protein adiponectin, which circulates in the plasma and is important in glucose and lipid metabolism [21]. *Adipoq* is expressed in white adipose tissue and is abundantly present in human plasma [22]. Low levels of adiponectin are associated with diabetes and there is a negative correlation between adiponectin and glucose levels in plasma [21], [23], [24]. It was reported that adiponectin activates three downstream pathways: AMPK phosphorylation, PPAR-α and p38-Mitogen-Activated Protein Kinase (MAPK) in liver and skeletal muscle, where it has the highest expression [25], [26].

The function of adiponectin in the brain is poorly understood, however, adiponectin receptors have been detected in the brain [27]. In particular, Yamauchi et al. (2003) demonstrated that the adiponectin receptors *AdipoR1* and *AdipoR2* are expressed in the hypothalamus to mediate glucose uptake by adiponectin [27]. Adiponectin receptors are expressed ubiquitously throughout the body [28]. Adiponectin was reported to mediate increased AMP kinase activity in the hypothalamus and stimulate food consumption [27], [28]. It has been shown that in leptin-deficient obese mice, adiponectin acts in the brain to reduce plasma glucose by 71%, insulin by 52%, triglycerides by 17% and total cholesterol by 29% [21], [29].

Currently, controversies concern adiponectin expression in the brain. In rats, the level of adiponectin was increased in the cerebrospinal fluid (CSF), but not in the plasma after intravenous injection [21], suggesting that the brain might be an important target for this hormone. According to Spranger et al. (2006), adiponectin is not expressed in the CNS [30]. The authors failed to detect adiponectin in human CSF samples and also did not find evidence of adiponectin crossing the blood brain barrier (BBB) [30]. However, brain endothelial cells express adiponectin receptors [30]. Nevertheless, several studies reported that adiponectin exists in human CSF at a 0.1% of serum concentration [31], [32].

Aside from direct effects via central receptors, adiponectin may also act through cell adhesion molecules. Interestingly, it has been proposed that adiponectin binds to the T-cadherin molecule [33]. T-cadherin can actively bind to some forms of adiponectin, suggesting its role as a possible part of a more complex signaling structure [29], [33]. T-cadherin belongs to a large family of proteins involved in calcium mediated cell–to-cell interactions and extracellular signaling [33]. The function of T-cadherin in the adiponectin system remains to be elucidated [29]. Intriguingly, here we observed that increased expression of the *Adipoq* gene occurred simultaneously with the up-regulation of the predicted *Cdh3* gene in the cerebellum. *Cdh3* is a P-cadherin gene which is overexpressed in the majority of pancreatic cancer, but not in healthy cells [34]. *Cdh3* is a novel tumor-associated antigen, which can be used in cancer immunotherapy [34]. Taking into consideration that experiments in mice suggest a role of *AdipoR2* in pancreatic islet cell maintenance [22], [29], there might be some connection between *Cdh3* and *Adipoq* functions in the process of developing pancreatic cancer.

Among other functions adiponectin is also implicated in regulation of blood pressure. Tanida et al. (2007) reported that adiponectin decreases blood pressure and sympathetic nerve activity in rats in a dose-dependent manner [35].

Interestingly, it was recently shown that adiponectin might be implicated in the pathophysiology of autism [36]. Mori and colleagues showed that serum levels of adiponectin in subjects with autism were significantly lower than those of normal controls [36]. Thus, we can conclude that functions of adiponectin are diverse and its specific role in the brain remains to be further investigated.

### Stress up-regulates the expression of prolactin receptor (PRLR)

The prolactin receptors are abundant in most tissues, with the highest expression in the liver, mammary glands, adrenal glands and hypothalamus [37]. Our results demonstrated that chronic restraint stress results in a two-fold increase in expression of prolactin receptor mRNA in the cerebellum. These findings are in consistency with previous results reporting that restraint stress in water causes up-regulation of the PRLR in the brain, specifically in the choroid plexus [38]. There were no significant differences in expression of PRLR in hippocampus and prefrontal cortex after two weeks of stress. However, recovery from stress altered the response in those two regions. The expression of PRLR in the hippocampus diminished significantly, whereas it was elevated in the prefrontal cortex. Thus, our results suggest a delayed response to mild restraint stress in the hippocampus and prefrontal cortex.

Prolactin (PRL) is a hormone produced by the pituitary, which is closely associated with the stress response [37], [39]. This hormone might play a role in emotional responses and HPA axis reactivity [40]. Evidence in a rat model suggests that prolactin is a neuromodulator of behavioural and neuroendocrine stress-responses, since it has central actions as an endogenous anxiolytic and anti-stress agent [40]. PRL functions are also implicated in reproduction, development, metabolic and immune processes, brain function and behaviour [37]. PRL levels may also increase in response to restraint stress and move from the blood circulation to the CSF, where it acts on the central nervous system [38]. These authors also demonstrated that circulating PRL causes prolactin receptor expression in the hypothalamus, suggesting a preventive role against stress-induced hypocalcemia and ulcerogenesis [38]. To regulate metabolic processes, PRL and PRLR are produced in human adipose tissue [41]. Since examination of adipose tissue from rodents and murine pre-adipocyte cell lines failed to detect PRL expression and release, expression of PRL in tissues other than the pituitary may be unique to humans and primates [37].

**Figure 11:**
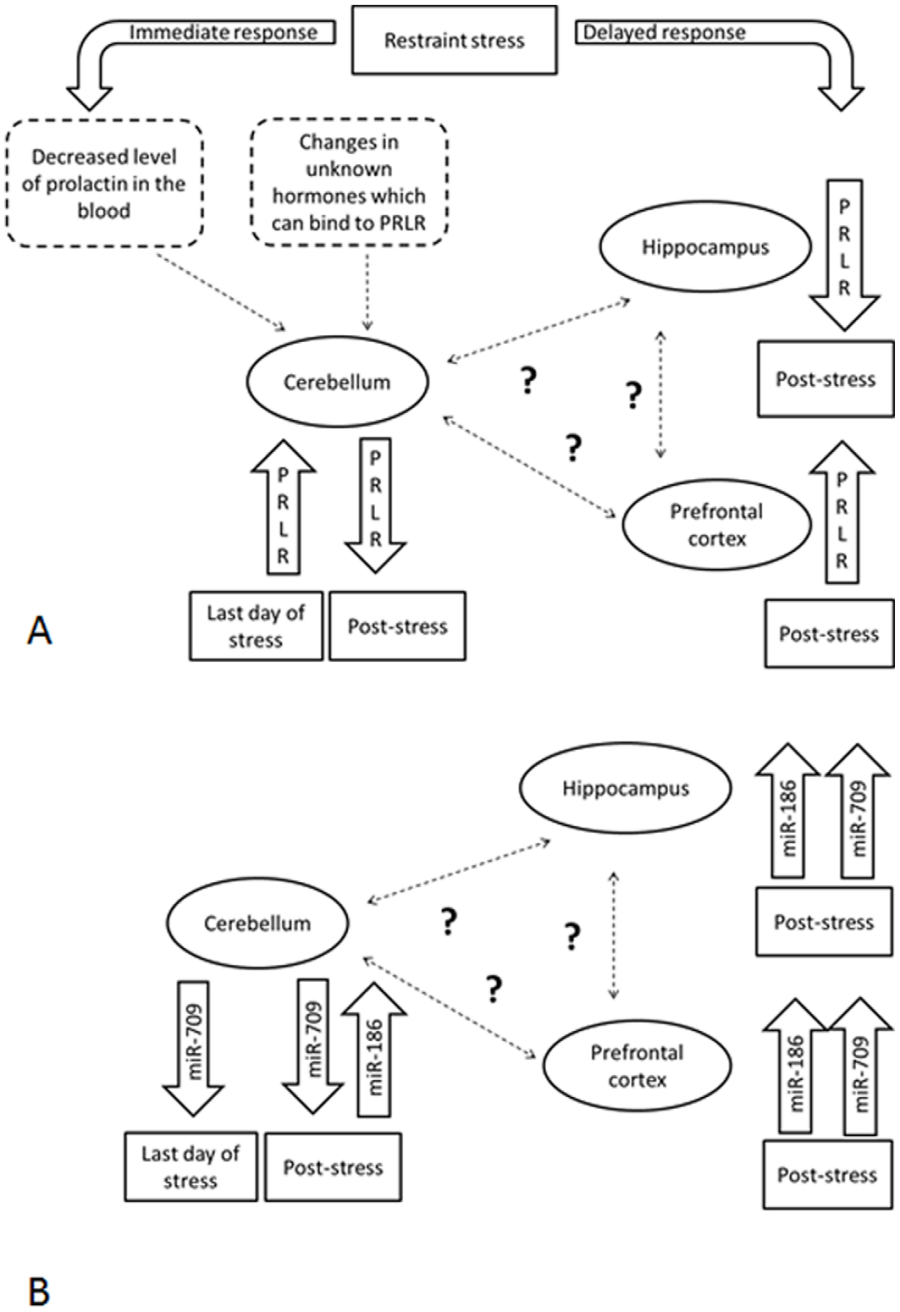
Hypothetical scheme of the stress response in the brain. A: Changes in PRLR expression. B: Changes in miRNA expression. We hypothesize that restraint stress causes a different response in cerebellum and other brain regions, such as hippocampus and prefrontal cortex. There might be an immediate response in the cerebellum reflected by an increase in the level of prolactin receptors after two weeks of stress. After the recovery from stress the expression of PRLR returns to normal in cerebellum, but is down-regulated in hippocampus and up-regulated in prefrontal cortex. Negative correlation between prolactin and its receptors [42] suggests that levels of prolactin in the cerebellum may decrease after stress. Alternatively, changes in expression levels of PRLR may occur in response to changes of other hormones or cytokines that bind to PRLR. Similarily, immediate responses in the cerebellum could be reflected in a decrease of miR-709 levels after two weeks of stress. After recovery from stress the expression of miR-709 is up-regulated in hippocampus and prefrontal cortex.

There is a negative correlation between prolactin and its receptors [42], suggesting that levels of prolactin in the cerebellum may increase after stress. Alternatively, changes in expression levels of PRLR may occur in response to changes of other hormones or cytokines that bind to PRLR. One could argue that on the first day of stress expression level of PRLR is elevated along with enhanced levels of anxiety. As illustrated in Figure 11, we propose that animals habituate to stress over time along with overexpression of PRLR that occurs as a result of the organism's stress response. Also, there might be an anatropic relation between PRLR levels in hippocampus and prefrontal cortex. It would be interesting to investigate connection between different brain regions in this regard, and find pathways that are involved in the regulation of stress response and role of PRLR in this process.

### Age up-regulates ephrin B3 and GABA A4 receptors

#### Ephrin B3 receptors

Interestingly, the present results show up-regulation of *Efnb3* expression in both stress and control animals after two weeks in comparison to stress and controls after four weeks. These observations suggest an age-dependent increase in *Efnb3* expression.

Ephrin receptors represent a large family of receptor protein tyrosine kinases, which play a crucial role in neuronal survival, axonal pathfinding and establishing neuron-target connections during embryonic development [43]. The expression of ephrin receptors in the CNS is higher in embryos than in adults and differs depending on the type of receptors [44], [45]. For instance, an abundance of ephrin A receptors was shown to be expressed in the adult CNS, while expression of only a few ephrin B receptors was found in the adult brain [43]. There is a controversy about the expression of the *Efnb3* receptor in the adult brain, although *Efnb3* expression was reported in several adult mouse tissues, including whole brain and adult rat spinal cord [43], [46], [47]. The results of Willson et al. (2006) demonstrated that *Efnb3* is abundantly expressed throughout the adult rat brain, with the most prominent expression in the cerebellum, suggesting that its functional role expands beyond embryonic development [43]. Thus, ephrin B3 receptors and their ligands might play a role in maintaining formed axonal connections and synapses, as well as regulate synaptic plasticity in the mature nervous system [43].

#### Gabra4 receptors

Similarly to ephrin B3, the expression of GABA (A) receptor 4 was also up-regulated in two and four week groups of control animals. γ-Aminobutyric acid (GABA) receptors are signaling proteins that represent the major inhibitory neurotransmitter receptors in the central nervous system [48]. It was reported that the GABA (A) receptor 4 gene (*Gabra4*) could contribute to autism susceptibility in humans [49], [50]. Chugani et al. (2001) have observed age-related changes in the distribution of the GABA (A) receptors in the brain of epileptic children [51]. Their results demonstrated the highest expression of GABA receptors in the brain at the youngest age measured (2 years), which decreased exponentially with age [51].

Age-dependent differences were also reported for the distribution across the brain of various GABA (A) receptor subunits in rats [52], [53], [54]. Studies by Laurie et al. (1992) examined the embryonic and postnatal expression of 13 GABA (A) receptor subunit genes in the rat CNS, showing that each subunit exhibits a unique regional and temporal developmental expression profile [52]. All of these data suggest the importance of GABA receptors during development and aging.

### Chronic restraint stress changes miRNA expression in the cerebellum

There were also changes in miRNA molecules whose functions are not well known yet. In particular, miR-186 and miR-709, which changed significantly in response to stress, do not belong to those which are abundantly expressed in the brain, and particularly in the cerebellum. There is a lack of data on the expression of these miRNAs. Also, their functions and verified targets are still unknown.

#### Changes in miR-186 expression

Our study revealed that miR-186 can target *Eps15* in mammalian cells. miR-186 was reported to be expressed by postnatal oligodendrocyte lineage cells [55]. Nowadays there is no agreement on the specific rules of target recognition by miRNA. Some authors suggest that it requires a perfect match between the seed sequence and the miRNA binding site [56], while others speculate that a nearly perfect match in the seed sequence is enough for target recognition [57]. We have found that for the efficient regulation of a target gene (*Eps15*) the seed sequence miR-186 can have one mismatch.

Epidermal growth factor (EGF) receptor pathway substrate 15 gene (*Eps15*) was first identified as an endogenous substrate for the EGF1 receptor kinase [58]. *Eps15* is believed to have an important role in vesicular traffic, but its exact function is still unknown [59]. There is some evidence that *Eps15* may play a role in the clathrin-mediated endocytosis of synaptic vesicle membranes [60]. It was shown that *Eps15* is concentrated in the presynaptic nerve terminals in rat brain, suggesting a role in the molecular rearrangement of the clathrin coats [60].

#### Changes in miR-709 expression

*Nab1* is a theoretically predicted target for miR-709. Although there was a trend for the decrease of *Nab1* expression upon transfection of miR-709, the difference was not significant. Thus, we cannot unambiguously confirm that *Nab1* is a target of miR-709.

miR-709 was reported to be upregulated in response to X-ray-DNA damage in the germline in mouse testes, and in turn, downregulates BORIS (Brother of the Regulator of Imprinted Sites) to counteract aberrant DNA hypomethylation [61]. It was shown that miR-709 may impact the genes involved in cytoskeletal functions [62]. Zhang et al. (2009) reported that methyl-CpG binding protein 2 was the common predicted target for miR-709. Thus, miR-186 and miR-709 may trigger a cascade of molecular reactions which are important in the regulation of the stress response.

### Conclusion

The present study showed that mild chronic stress results in molecular changes in the brain on the genetic and epigenetic levels. The results suggests that even mild chronic stress can cause long-lasting changes in motor function in the intact brain and recovery from brain injury through genomic and epigenomic pathways.

## Materials and Methods

### Ethics statement

All procedures were performed in accordance with the Canadian Council for Animal Care guidelines and approved by the local animal welfare committee (protocol #1007). All data are MIAME compliant.

### Animals

Thirty-five male adult Long-Evans hooded rats, approximately four months old (weighing approximately 460 g at the beginning of the study), from Charles River Laboratories International Inc. (Wilmington, MA, US) were used. Animals were habituated to the local environment for a period of three weeks prior to the start of the experiment. The rats were housed in pairs in standard polycarbonate shoebox cages under a 12 h light/day cycle with lights on at 7:30 AM.

### Experimental groups

Animals were randomly assigned to one of the following experimental groups: Two weeks of daily restraint stress (2WSTRESS, n = 6), two weeks naive controls (2WCONTROL, n = 6), two weeks of daily restraint stress + two weeks of recovery from stress (4WSTRESS, n = 6), four weeks naive controls (4WCONTROL, n = 6). A separate group of rats was trained and tested in the skilled reaching task to confirm restraint stress-induced motor impairments (n = 11).

### Time course

**Figure 12:**
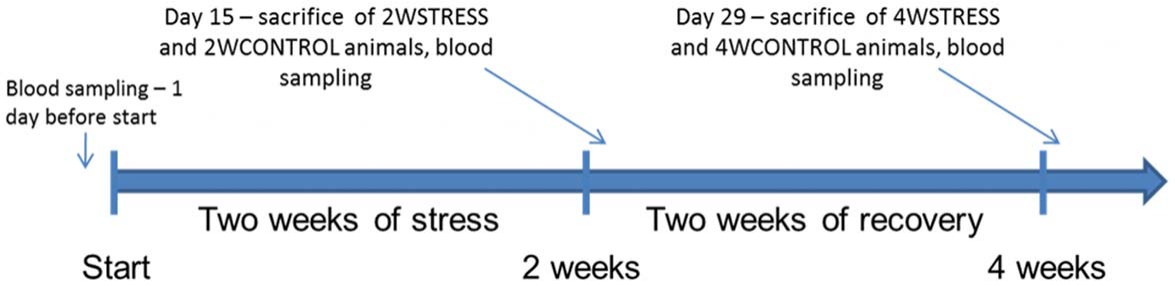
Time course of the experimental manipulations. 24 animals were randomly assigned to one of the following experimental groups: two weeks of daily restraint stress (2WSTRESS, n = 6), two weeks naive controls (2WCONTROL, n = 6), two weeks of daily restraint stress + two weeks of recovery from stress (4WSTRESS, n = 6), four weeks naive controls (4WCONTROL, n = 6). Animals were sacrificed immediately after stress (2 weeks), or after two weeks of recovery from stress (4 weeks).

The 2WSTRESS group was subjected to 14 days of restraint stress, while the 4WSTRESS group received two weeks of recovery after two weeks of restraint treatment. Stressed and respective control animals were sacrificed immediately after the last day of stress and after 14 days of recovery from stress (Fig. 12). Blood samples were collected at baseline, on the first and last day of stress treatment, and on the last day of recovery after stress.

### Physiological manipulations and testing

#### Restraint stress

Animals were placed individually into transparent Plexiglas tubes (diameter 8 cm, length 20 cm) for 20 minutes daily in the morning hours. The tube maintained the animal in a standing position. Small holes in the front of the tube allowed for ventilation. Restraint took place each day at the same time in the morning hours and in a room different from the colony room.

#### Blood sampling

Blood samples were collected between 08:30AM and 11:00AM in order to minimize effects of plasma corticosterone circadian variations [63]. Samples were collected approximately 30 minutes after initiation of restraint stress [64]. Rats were anesthetized with 4% isoflurane in 30% oxygen. Syringes and butterfly catheters were rinsed with heparin prior to use for blood sampling. An average of 0.6 ml of blood was collected from the tail vein using a butterfly catheter. The sample was centrifuged at 4000 rpm for 10 minutes. After centrifugation plasma was collected and stored at −20°C.

#### Corticosterone enzyme immunoassay (EIA)

Quantification of corticosterone in plasma samples was performed with a Corticosterone EIA Kit according to the manufacturer's instructions (Cayman Chemical Company, Ann Arbor, MI, USA). Calculations and data analysis were performed using the original spreadsheet supplied by Cayman.

#### Behavioural testing

Skilled movement performance in rats was assessed using a pellet reaching task according to earlier descriptions [65]. Briefly, animals were trained to extend their forelimbs to reach for 45 mg food pellets through a vertical opening in the middle of the front wall of a clear Plexiglas box. Rats were trained for three weeks daily to achieve asymptotic levels of baseline success rates. Daily tests in skilled reaching continued while animals were exposed to 14 days of restraint. Each training and test session required the rats to reach for 20 food pellets. A successful reach was recorded if an animal grasped a food pellet on the first attempt and withdrew the paw with the pellet through the slit to consume the food (Metz & Whishaw, 2000). Percent reaching success was calculated by counting the number of successful reaches divided by the number of pellets given in each session (20) multiplied by 100.

### Brain tissue dissection and RNA extraction

The rats were deeply anaesthetized with pentobarbital (WDDC, Edmonton, AB, Canada). After vital signs discontinued animals were rapidly decapitated. Brains were rapidly removed. The brains of four animals from each group were used for dissection of cerebellum, prefrontal cortex and hippocampus. The brains of two animals from each group were flash-frozen. Dissected cerebellum was used for DNA/RNA extractions.

### RNA extractions

The RNA isolation was performed using TRIzol reagent (Invitrogen, Carlsbad, CA, USA) according to manufacturer's instructions. The samples were treated with DNase I (Invitrogen, Carlsbad, CA, USA) according to manufacturer's instructions and stored at -80°C. DNA and RNA were dissolved in RNase-free water (NEB, Ipswich, MA, USA).

### Plasmid DNA purification

Plasmid DNA was purified from overnight culture (20 ml LB medium supplemented with corresponding antibiotics) using the QIAprep Spin Miniprep Kit (Qiagen, Valencia, CA, USA).

### cDNA synthesis

cDNA synthesis was performed using the RevertAid™ H Minus First Strand cDNA Synthesis Kit (Fermentas, Barlington, ON, Canada). For the reaction 4.6 µg of total RNA, 1 unit of Ribolock RNase inhibitor, 10 mM dNTPs, oligo(dT_18_) primers, and 5× reaction buffer were used. The mix was incubated for 1 h at 42°C and heat inactivated for 5 min at 70°C.

### miRNA microarray expression analysis

Tissue from three animals per group was used for miRNA expression analysis. Total RNA was extracted from the cerebellum. The miRNA microarray analysis was performed by LC Sciences (Houston, TX, USA; www.lcsciences.com). The assay used from 2 to 5 µg of the total RNA sample, which was fractionated by size using a YM-100 Microcon centrifugal filter (Millipore, Billerica, MA, USA) and the small RNAs (<300 nt) isolated were 3′-extended with a poly(A) tail using poly(A) polymerase. An oligonucleotide tag was then ligated to the poly(A) tail for later fluorescent dye staining. Two different tags were used for the two RNA samples in dual-sample experiments.

Hybridization was performed overnight on a µParaflo microfluidic chip (LC Sciences, Houston, TX, USA) using a micro-circulation pump. On the microfluidic chip, each detection probe consisted of a chemically modified nucleotide coding segment complementary to the target miRNA (from miRBase, Welcome Trust Sanger Institute, Cambridge, UK; http://microrna.sanger.ac.uk/sequences/) or control RNA and a spacer segment of polyethylene glycol to extend the coding segment away from the substrate. The detection probes were made by *in situ* synthesis using PGR (photogenerated reagent) chemistry. The hybridization melting temperatures were balanced by chemical modifications of the detection probes. Hybridization used 100 µL 6× SSPE buffer (0.9 M NaCl, 60 mM Na_2_HPO_4_, 6 mM EDTA, pH 6.8) containing 25% formamide at 34°C. After hybridization detection fluorescence labeling using tag-specific Cy3 and Cy5 dyes was performed. Hybridization images were collected using a laser scanner (GenePix 4000B, Molecular Devices, Sunnyvale, CA, USA) and digitized using Array-Pro image analysis software (Media Cybernetics, Bethesda, MD, USA). Data were analyzed by first subtracting the background and then normalizing the signals using a LOWESS filter10 (Locally-weighted Regression). For two-color experiments, the ratio of the two sets of detected signals (log_2_ transformed, balanced) and p-values of the t-test were calculated; differentially detected signals were those with less than 0.01 p-values (information provided by Jason Mulcahey, LC Sciences).

### mRNA microarrays

Three RNA samples out of four per group were chosen for microarray analysis based on RNA quality and concentration. The mRNA microarray analysis was performed by Genome Quebec (Montréal, Quebec, Canada; www.genomequebecplatforms.com). The sample used 250 ng of starting total RNA to hybridize 750 ng of biotin labeled cRNA on the RatRef-12 array. Amplification was performed using the Illumina TotalPrep RNA Amplification kit (Ambion, Austin, TX, USA). Illumina's Gene Expression system uses a “direct hybridization” assay whereby biotin-labeled samples are hybridized individually to an array. Following hybridization the transcripts were detected using Cy3 conjugated streptavidin and scanned. The signal intensities generated provide an indication of the absolute abundance of transcripts within that population. The signal intensities generated from separate arrays can be compared as an indication of the differences between the two sample populations (Illumina, San Diego, CA, USA; www.illumina.com).

### Semi-quantitative reverse transcription PCR (sqRT-PCR)

The sqRT-PCR was performed by using Taq DNA Polymerase (Fermentas, Burlington, ON, Canada), specific primers and carried out on an Eppendorf Mastercycler PCR machine (Eppendorf, Hamburg, Germany). Primers were designed using Primer3 v. 0.4.0 software [66] and synthesized by Integrated DNA Technologies (San Diego, CA, USA). Each reaction contained 1 µl of cDNA, 0.5 µl of 10 µM forward and reverse primers, 2.5 µl of 10× Taq Buffer, 2.5 µl of 2 mM dNTPs, and 0.25 µl of Taq DNA polymerase (5 U/µl) in a total volume of 25 µl. For PCR conditions and primer sets see (Table S1). PCR conditions where empirically determined for each set of primers so that the concentration of PCR product was below saturation point. Agarose gel electrophoresis was carried out in 1× TAE buffer using a 1% agarose gel with ethidium bromide. The amplified product was visualized under UV light and quantified using ImageQuant 5.2 software (GE Healthcare, Piscataway, NJ, USA) and normalized to *Gapdh* gene expression.

### Quantitative real time PCR (qRT-PCR) with miRNAs

#### RNA purification

Total RNA was purified from 100–150 mg of flash-frozen brain tissues using TRIzol reagent (Invitrogen, Carlsbad, CA, USA) according to manufacturer's instructions. Following purification, 5 µg of each RNA sample were treated with 2 U of DNase I (Fermentas, Burlington, ON, Canada) at 37°C for 30 min. After treatment DNase I was heat-inactivated (65°C for 10 min) in the presence of 5 mM EDTA, pH 8.0.

#### cDNA synthesis

cDNA for qRT-PCR was synthesized as previously described [67] using iScript Select cDNA Synthesis Kit (Bio-Rad Laboratories Ltd., Mississauga, Ontario, Canada) according to manufacturer's instructions. Briefly, 100 ng of DNase I treated RNA were reverse-transcribed with 2 µl of iScript reverse transcriptase and RNase inhibitor protein, and 100 nM of each RT primer (Table 1), in the total volume of 40 µl. Reaction was carried out at 42°C for 30 min. Reverse transcriptase was heat-inactivated at 85°C for 5 min.

#### qRT-PCR

qPCR was done as previously described [67] using SsoFast EvaGreen Supermix (Bio-Rad Laboratories Ltd., Mississauga, Ontario, Canada) and CFX96 system (Bio-Rad Laboratories Ltd., Mississauga, Ontario, Canada). Briefly, qRT-PCR was carried out in a total volume of 20 µl, in the presence of 10 µl of 2× SsoFast EvaGreen Supermix, 0.5 µl of 10 mM corresponding forward and reverse primer (Table 1), and 1 µl of each cDNA sample. Cycling conditions for qPCR: enzyme activation −95°C for 30 sec, denaturation −95°C for 5 sec, annealing/extension −60°C for 5 sec (45 cycles), melting curve – 65–95°C (5 sec/step). The Ct values (the threshold cycles) were calculated with the CFX Manager 2.0 software. All miRNA expression levels were normalized to the RNU-6 snRNA expression.

### qRT-PCR with mRNA

#### RNA purification

After purification with TRIzol reagent (Invitrogen, Carlsbad, CA, USA) and DNase I treatment, total RNA was additionally cleaned up with RNAeasy RNA purification kit (QIAGEN) according to manufacturer's instructions.

#### cDNA synthesis

cDNA was synthesized using iScript Select cDNA Synthesis Kit (Bio-Rad Laboratories Ltd., Mississauga, Ontario, Canada) according to manufacturer's instructions. Briefly, 500 ng of DNase I treated RNA were reverse-transcribed with 2 µl of iScript reverse transcriptase, RNase inhibitor protein, and oligo(dT18), in the total volume of 40 µl. Reaction was carried out at 42°C for 90 min. Reverse transcriptase was heat-inactivated at 85°C for 5 min.

#### qRT-PCR

qRT-PCR was done using SsoFast EvaGreen Supermix (Bio-Rad Laboratories Ltd., Mississauga, Ontario, Canada) and CFX96 system (Bio-Rad Laboratories Ltd., Mississauga, Ontario, Canada) according to manufacturer's instructions. Briefly, qRT-PCR was carried out in a total volume of 20 µl, in the presence of 10 µl of 2× SsoFast EvaGreen Supermix, 0.5 µl of 10 mM corresponding forward and reverse primer (Table 1), and 1 µl of each cDNA sample. Cycling conditions for qPCR: enzyme activation −95°C for 30 sec, denaturation −95°C for 5 sec, annealing/extension −51°C for 5 sec (45 cycles), melting curve – 65–95°C (5 sec/step). The Ct values were calculated with the CFX Manager 2.0 software. All mRNA expression levels were normalized to the β-Actin mRNA expression.

### miRNA target prediction

miRNA targets for further analysis were predicted using basic seed-based algorithms [68] from the Targetscan database (Whitehead Institute for Biomedical Research, Cambridge, MA, USA; www.targetscan.org). Predicted targets of a miRNA family were calculated as published earlier [69] and sorted by total context score. The total context score was based on the following features: site-type contribution, 3′ pairing contribution, local AU contribution, and position contribution [69].

### Cloning

The 3′- untranslated regions (UTR) of the *Eps15* gene (NM_001009424) with a seed sequence for the miR-186 and *Nab1* (NM_022856) genes with a binding site for miR-709 (see Fig. S5) were amplified by PCR. They were then cloned into pGL3-Promoter vectors downstream of the Luciferase coding sequence, resulted in pFN4 (*Eps-15* 3′ UTR) and pFN7 (*Nab1* 3′ UTR) plasmids. The PCR mix contained 10 µl of 5× GC buffer, 5 µl of 2 mM dNTPs, 1 µl of 10 µM forward and reverse primers (see,Table S12), 1 µl of cDNA, and 0.5 µl of Phusion® High-Fidelity DNA Polymerase (NEB, Ipswich, MA, USA) in a total volume of 50 µl. The PCR conditions were: initial denaturation −30 sec at 98°C, 25 cycles with denaturation −10 sec at 98°C, annealing −30 sec at 63°C, polymerization −30 sec at 72°C, and final extension 10 min at 72°C.

### Binding site mutagenesis

To create mismatch controls the pFN4 plasmid was mutated with primers MS026 and MS027, and pFN7 with primers MS044 and MS055 that carry mutated seed sequences. PCR fragments were generated with Phusion® High-Fidelity DNA Polymerase (see section ***Cloning***), extracted from the 1% agarose gel with QIAquick Gel Extraction Kit (Qiagen, Valencia, CA, USA) and self-ligated using T4 DNA ligase (Fermentas, Burlington, ON, Canada). Mutated plasmids (pFN4mut and pFN7mut) were screened by colony PCR with corresponding primers: pFN4mut – MS028 and AG264 (see Fig. S6), pFN7mut – MS046 and AG264. Original and mutated plasmids were confirmed by DNA sequencing.

### Cell culture and Luciferase Reporter Assay

Human embryonic kidney HEK-293 cells were maintained in DMEM (Invitrogen, Carlsbad, CA, USA), supplemented with 10% fetal bovine serum, penicillin (100 U/ml) at 37°C in a 5% CO_2_ atmosphere. HEK293 cells were co-transfected in 24-well plates with the pGL3 vector (with Firefly luciferase) or tested construct, precursor miRNA, and control Renilla luciferase pRL-TK vector (Promega, Madison, WI, USA), using the Lipofectamine 2000 reagent according to the manufacturer's protocol (Invitrogen, Carlsbad, CA, USA). Twenty-four hours after transfection, 1× Passive Lysis Buffer (Promega, Madison, WI, USA) was added to the transfected cells. Renilla and Firefly luciferase activities were measured using the dual-luciferase reporter assay system with Stop & Glow Reagent (Promega, Madison, WI, USA) according to the manufacturer's instructions. A similar culture experiment was performed using another cell line, human breast cancer MCF-7 cells. Each cell line was tested in triplicate and reproduced twice in independent experiments.

### Data analysis and statistics

Statistical analysis was performed using Microsoft Excel Analysis ToolPak (Microsoft Corp., Redmond, WA, USA). All data are presented as the mean +/− standard deviation. Details of each type of analysis are provided in the following.

#### Behavioural tests

Data were analyzed using repeated measures analyses of variance (ANOVA) using Time as a variable. Differences between baseline and stress periods were determined post-hoc using paired Student's t-tests.

#### mRNA and miRNA microarray analysis

mRNA microarray analysis was performed using FlexArray 1.4.1 software [70]. The data analysis was performed using the lumi Bioconductor package [71], which was used for the pre-processing and normalizing of Illumina microarray data. Background correction was performed using Robust Multichip Average (RMA) background adjustment [72], [73], [74]. Log_2_ data were normalized using Quantile normalization. A two-sample student's t-test was run to compare gene expression in different groups. Data were plotted using volcano plots of p-values. The list of genes 2-fold up- or down-regulated with a p-value of ≤0.05 was generated.

The miRNA microarray data analysis was performed by LC Sciences (Houston, TX, USA). Data analysis included the determination of detectable signals, calculation of signal intensities, and calculation of differential ratios. The data analysis process began with background subtraction, Cy3/Cy5 channel normalization, detectivity determination, and then p-value calculation for the determination of differential significance. Multiple sample analysis involved normalization, data adjustment, t-test/ANOVA analysis, and clustering.

qRT-PCR data analysis was done using Pfaffl method [75]. Data are presented as a fold change of each mRNA from the tissues of stressed animals relative to non-stressed controls corrected for internal standard.

## Supporting Information

Figure S1 **qRT-PCR data of** ***Prlr*** **expression.**
(TIF)

Figure S2 **qRT-PCR data of** ***Adipoq*** **expression.**
(TIF)

Figure S3 **qRT-PCR data of miR-186 expression.**
(TIF)

Figure S4 **qRT-PCR data of miR-709 expression.**
(TIF)

Figure S5 A: Putative binding site of mir-186 in *Eps15* 3′UTR. B: Putative binding site of mir-709 in *Nab15* 3′UTR. The seed sequence is represented in blue, while the mutated seed sequence is shown in red.
(TIF)

Figure S6 **Schematic illustration of binding site mutagenesis.** The PCR fragment with a mutated binding sequence was obtained by inverse PCR with corresponding primers from the original pFN4 plasmid. The miR-186 binding sequence (highlighted in red) was substituted with the mutated sequence (adenine was substituted by guanine, while thymine was substituted by guanine).
(TIF)

Table S1 **qRT-PCR data of** ***Prlr*** **expression in hippocampus.**
(DOC)

Table S2 **qRT-PCR data of** ***Prlr*** **expression in prefrontal cortex.**
(DOC)

Table S3 **qRT-PCR data of** ***Adipoq*** **expression in hippocampus.**
(DOC)

Table S4 **qRT-PCR data of** ***Adipoq*** **expression in prefrontal cortex.**
(DOC)

Table S5 **Functional annotation clustering analysis of target genes.**
(DOC)

Table S6 **qRT-PCR data of miR-186 expression in cerebellum.**
(DOC)

Table S7 **qRT-PCR data of miR-709 expression in cerebellum.**
(DOC)

Table S8 **qRT-PCR data of miR-186 expression in hippocampus.**
(DOC)

Table S9 **qRT-PCR data of miR-709 expression in hippocampus.**
(DOC)

Table S10 **qRT-PCR data of miR-186 expression in prefrontal cortex.**
(DOC)

Table S11 **qRT-PCR data of miR-709 expression in prefrontal cortex.**
(DOC)

Table S12 **Primers and PCR conditions for sq-RT-PCR, qRT-PCR, and cloning.**
(DOCX)
