# Supplementary figures and images for: Genomic and Epigenomic Responses to Chronic Stress Involve miRNA-Mediated Programming
Source: PLoS One. 2012 Jan 24;7(1):e29441. doi: 10.1371/journal.pone.0029441 (PMC3265462; doi:10.1371/journal.pone.0029441)

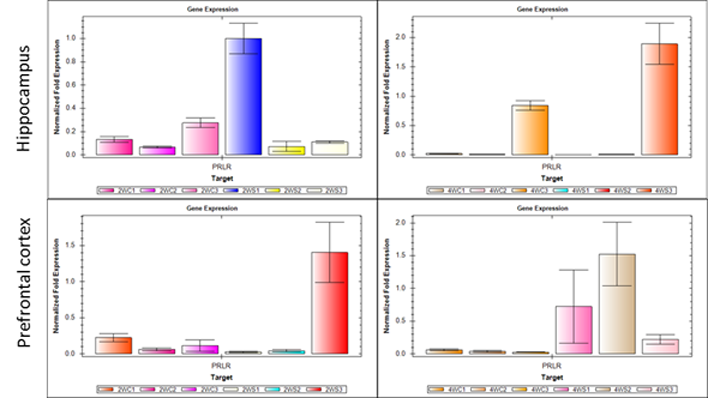

Supplement: Figure S1 — qRT-PCR data of Prlr expression. (TIF) [file pone.0029441.s001.tif]

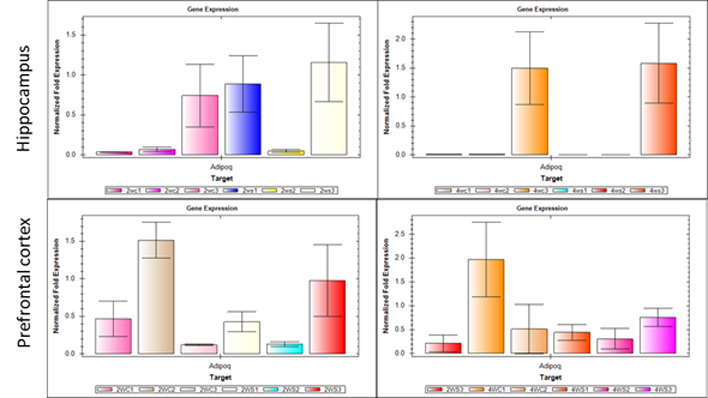

Supplement: Figure S2 — qRT-PCR data of Adipoq expression. (TIF) [file pone.0029441.s002.tif]

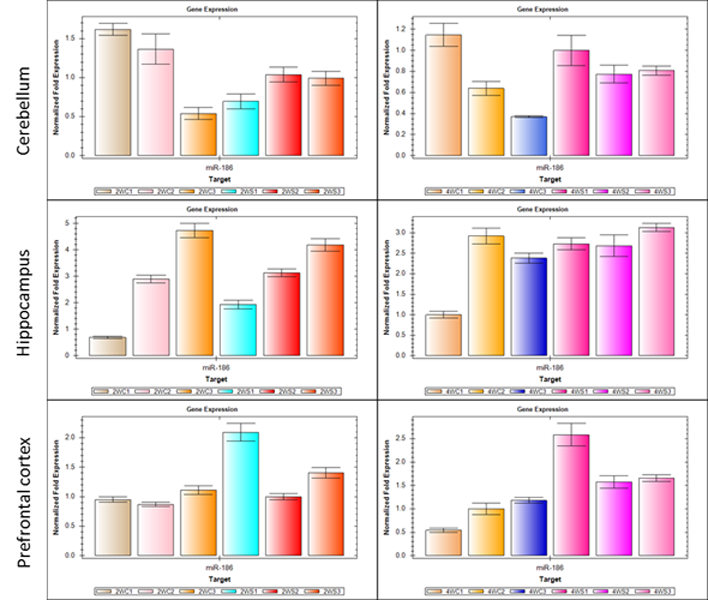

Supplement: Figure S3 — qRT-PCR data of miR-186 expression. (TIF) [file pone.0029441.s003.tif]

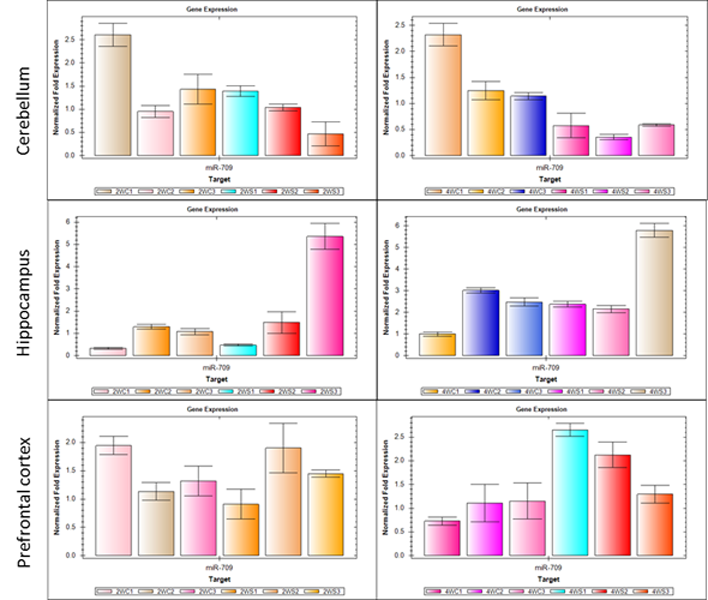

Supplement: Figure S4 — qRT-PCR data of miR-709 expression. (TIF) [file pone.0029441.s004.tif]

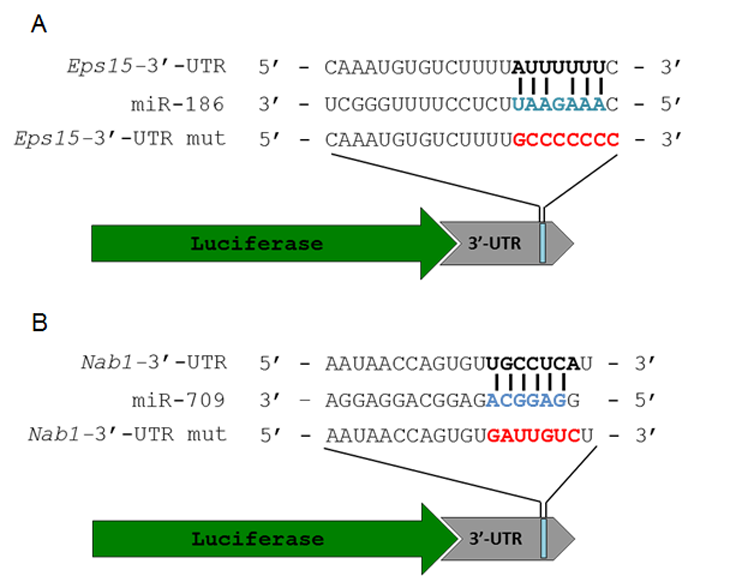

Supplement: Figure S5 — A: Putative binding site of mir-186 in Eps15 3′UTR. B: Putative binding site of mir-709 in Nab15 3′UTR. The seed sequence is represented in blue, while the mutated seed sequence is shown in red. (TIF) [file pone.0029441.s005.tif]

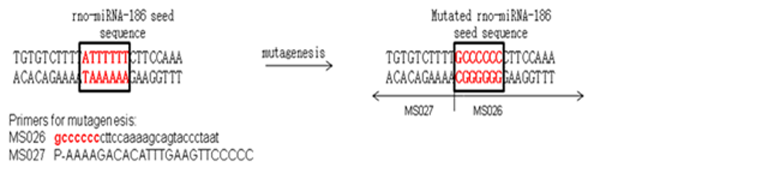

Supplement: Figure S6 — Schematic illustration of binding site mutagenesis. The PCR fragment with a mutated binding sequence was obtained by inverse PCR with corresponding primers from the original pFN4 plasmid. The miR-186 binding sequence (highlighted in red) was substituted with the mutated sequence (adenine was substituted by guanine, while thymine was substituted by guanine). (TIF) [file pone.0029441.s006.tif]
