# Supplementary material for: Genomic and Epigenomic Responses to Chronic Stress Involve miRNA-Mediated Programming
Source: PLoS One. 2012 Jan 24;7(1):e29441. doi: 10.1371/journal.pone.0029441 (PMC3265462; doi:10.1371/journal.pone.0029441)
Supplement: Table S1 — qRT-PCR data of Prlr expression in hippocampus. (DOC) [file pone.0029441.s007.doc]

**Table S1.** Primers and PCR conditions for sq-RT-PCR, qRT-PCR, and cloning.

| *Gene/ Object* | *Primer* | *Sequence* | *Note* |
| --- | --- | --- | --- |
|  |  |  |  |
| Eps15 | for | AAATCTAGATAGTCCCAAAGAAAAGGATCCTGATATG | Initial denaturation - 98˚C for 30 sec, 30 cycles with initial denaturation 98˚C for 10 sec, annealing - 58˚C for 30 sec, polymerization - 72˚C for 30 sec, final extension -72˚C for 3 min. |
|  | rev | AAAGGCCGGCCGGCAAAGCTGTTTAATTAGGGTACTGC |  |
| Nab1 | for | AAATCTAGACTGAGATTCTCCCCCTGCTCTCTGG | Initial denaturation - 98˚C for 30 sec, 30 cycles with initial denaturation 98˚C for 10 sec, annealing - 58˚C for 30 sec, polymerization - 72˚C for 30 sec, final extension -72˚C for 3 min. |
|  | rev | AAAGGCCGGCCGGGATCCACGTCTATTAGACTGCAAGG |  |
| EPS15 | for | gcccccccttccaaaagcagtaccctaat | Initial denaturation - 98˚C for 30 sec, 30 cycles with initial denaturation 98˚C for 10 sec, annealing - 62˚C for 30 sec, polymerization - 72˚C for 30 sec, final extension -72˚C for 3 min. |
|  | rev | P-AAAAGACACATTTGAAGTTCCCCC |  |
|  | check | GGGGAACTTCAAATGTGTCTTTTgcc |  |
| NAB1 | for | GATTGTCTTCAGCTCCCAGACCA | Initial denaturation - 98˚C for 30 sec, 30 cycles with initial denaturation 98˚C for 10 sec, annealing - 60˚C for 30 sec, polymerization - 72˚C for 30 sec, final extention -72˚C for 3 min. |
|  | rev | P-ACACTGGTTATTAAGGCTTAATCTACGAT |  |
|  | check | GTAGATTAAGCCTTAATAACCAGTGTgat |  |
| ADIPOQ | for | ATGCTACTGTTGCAAGCGCTCC | Initial denaturation - 95˚C for 3 min, 31 cycles with initial denaturation 95˚C for 30 sec, annealing - 61.6˚C for 30 sec, polymerization - 72˚C for 30 sec. |
|  | rev | TCAGTTGGTATCATGGTAGAGA |  |
| Ephrinb3 | for | ATGGGGGGCCCCCATTTTGGGCC | Initial denaturation - 95˚C for 3 min, 32 cycles with initial denaturation 95˚C for 30 sec, annealing - 62˚C for 30 sec, polymerization - 72˚C for 30 sec. |
|  | rev | TCATACCTTGTAGTAGATGTTT |  |
| GABRA4 | for | ATGGTTTCTGTCCAGAAGGTAC | Initial denaturation - 95˚C for 3 min, 29 cycles with initial denaturation 95˚C for 30 sec, annealing - 61˚C for 30 sec, polymerization - 72˚C for 30 sec. |
|  | rev | TTACATTAGACTTTCTGATTTC |  |
| PRLR | for | ATGCCATCTGCACTTGCTTTCG | Initial denaturation - 95˚C for 3 min, 27 cycles with initial denaturation 95˚C for 30 sec, annealing - 60.8˚C for 30 sec, polymerization - 72˚C for 30 sec. |
|  | rev | TCAGTAGTCAAGTTCCCCTGCA |  |
| GAPDH | for | CAAGGTCATCCATGACAACTTTG | Initial denaturation - 95˚C for 3 min, 22 cycles with initial denaturation 95˚C for 30 sec, annealing - 55˚C for 30 sec, polymerization - 72˚C for 30 sec. |
|  | rev | GTCCACCACCCTGTTGCTGTAG |  |
| pGL3 | rev | GGTTACAAATAAAGCAATAGCATCACA |  |
| A2bp1 | for | AAAGGCCGGCCATGATAAAACCATTAAACAAACAAACAAAAAAC | Initial denaturation - 98˚C for 30 sec, 30 cycles with initial denaturation 98˚C for 10 sec, annealing - 60˚C for 30 sec, polymerization - 72˚C for 30 sec, final extension -72˚C for 3 min. |
|  | rev | AAAGGCCGGCCGGCCTCCCCTTTCACAGAATAAAATAATATAG |  |
| Creb5 | for | AAATCTAGAAAGGCATCGGTCAAACCTGGCC | Initial denaturation - 98˚C for 30 sec, 30 cycles with initial denaturation 98˚C for 10 sec, annealing - 60˚C for 30 sec, polymerization - 72˚C for 45 sec, final extension -72˚C for 3 min. |
|  | rev | AAAGGCCGGCCCTCTAAAATAGAATTTTTTTTAAACTATAGTGAGCG |  |
| MAP3K2 | for | AAATCTAGACAGCCAGCGTCCTCCACCCGCC | Initial denaturation - 98˚C for 30 sec, 30 cycles with initial denaturation 98˚C for 10 sec, annealing - 60˚C for 30 sec, polymerization - 72˚C for 45 sec, final extension -72˚C for 3 min. |
|  | rev | AAAGGCCGGCCTTTAGTTTACTATTATTATTAAACAAATTTAACCAAG |  |
| miR-186 | RT oligo | caccgttccccgccgtcggtgAGCCCA | Initial denaturation - 95˚C for 2 min, 45 cycles with initial denaturation 95˚C for 5 sec, annealing/polymerization - 61˚C for 5 sec; melt curve 65˚C to 95˚C, increment 0.5˚C for 5 sec. |
|  | for | CCCGCCCAAAGAATTCTCCTT |  |
|  | rev | gccgtcggtgAGCCCAAAA |  |
| miR-709 | RT oligo | caccgttccccgccgtcggtgTCCTCC | Initial denaturation - 95˚C for 2 min, 45 cycles with initial denaturation 95˚C for 5 sec, annealing/polymerization - 61˚C for 5 sec; melt curve 65˚C to 95˚C, increment 0.5˚C for 5 sec. |
|  | for | CGCCGGAGGCAGAGGCA |  |
|  | rev | gccgtcggtgTCCTCCTGC |  |
| RNU-6 | RT oligo | caccgttccccgccgtcggtgCGCTTC | Initial denaturation - 95˚C for 2 min, 45 cycles with initial denaturation 95˚C for 5 sec, annealing/polymerization - 61˚C for 5 sec; melt curve 65˚C to 95˚C, increment 0.5˚C for 5 sec. |
|  | for | CGCCctgcgcaaggatgac |  |
|  | Rev | ccgtcggtgCGCTTCACG |  |
| Adipoq | for | ATGTATCACTCAGCATTC | Initial denaturation - 95˚C for 2 min, 45 cycles with initial denaturation 95˚C for 5 sec, annealing/polymerization – 51˚C for 5 sec; melt curve 65˚C to 95˚C, increment 0.5˚C for 5 sec. |
|  | rev | CTGTTGGTTGTAGAAGAT |  |
| Prlr | for | GGACAGATGGAGGACTTC | Initial denaturation - 95˚C for 2 min, 45 cycles with initial denaturation 95˚C for 5 sec, annealing/polymerization – 52.4˚C for 5 sec; melt curve 65˚C to 95˚C, increment 0.5˚C for 5 sec. |
|  | rev | GGACATTCGTAGGTGGTT |  |
