# Supplementary material for: Genomic and Epigenomic Responses to Chronic Stress Involve miRNA-Mediated Programming
Source: PLoS One. 2012 Jan 24;7(1):e29441. doi: 10.1371/journal.pone.0029441 (PMC3265462; doi:10.1371/journal.pone.0029441)
Supplement: Table S2 — qRT-PCR data of Prlr expression in prefrontal cortex. (DOC) [file pone.0029441.s008.doc]

**Table S2. Functional annotation clustering analysis of target genes.**

| **Annotation cluster** | **Genes** | **Enrichment score** | **Fold Enrichment** | **P-value** |
| --- | --- | --- | --- | --- |
| **Positive regulation of macromolecule metabolic process** | TCF21, MSX1, ADIPOQ, CITED | 1.17 | 4.885 | 0.034 |
| **Protein complex assembly** | PRLR, OTC, ADIPOQ | 1.164 | 7.284 | 0.052 |
| **Cell adhesion** | CLDN3, ITGB6, CDH3 | 0.968 | 6.529 | 0.064 |
| **Receptor** | PRLR, OSMR, ITGB6, STRA6 | 0.737 | 3.528 | 0.072 |
