# Supplementary material for: Genomic and Epigenomic Responses to Chronic Stress Involve miRNA-Mediated Programming
Source: PLoS One. 2012 Jan 24;7(1):e29441. doi: 10.1371/journal.pone.0029441 (PMC3265462; doi:10.1371/journal.pone.0029441)
Supplement: Table S3 — qRT-PCR data of Adipoq expression in hippocampus. (DOC) [file pone.0029441.s009.doc]

**Table S3.** qRT-PCR data of *Prlr* expression in hippocampus.

| **Gene** | **Sample #** | **Sample name** | **C(t)** | | | **Average C(t)** | **St.dev.** | **Average C(t) and st. dev. from biological repeats** | |
| --- | --- | --- | --- | --- | --- | --- | --- | --- | --- |
| Prlr (Gene of interest) | 1 | 2WS1 | 30.57 | 30.31 | 30.81 | **30.56** | 0.25 |  |  |
|  | 2 | 2WS2 | n/a | 36.52 | 35.19 | **35.86** | 0.94 | 2WStress | |
|  | 3 | 2WS3 | 34.27 | 34.2 | 33.87 | **34.11** | 0.21 | **33.51** | **2.70** |
|  | 4 | 2WC1 | n/a | 35.19 | 35.68 | **35.44** | 0.35 |  |  |
|  | 5 | 2WC2 | 34.58 | 34.72 | 35.09 | **34.80** | 0.26 | 2WControl | |
|  | 6 | 2WC3 | 27.81 | 26.97 | 27.47 | **27.42** | 0.42 | **32.55** | **4.46** |
|  | 7 | 4WS1 | 33.87 | 33.91 | 34.63 | **34.14** | 0.43 |  |  |
|  | 8 | 4WS2 | 34.95 | 34.58 | 34.39 | **34.64** | 0.28 | 4WStress | |
|  | 9 | 4WS3 | 32.32 | 33.03 | 32.66 | **32.67** | 0.36 | **33.82** | **1.02** |
|  | 10 | 4WC1 | 33.35 | 34.02 | 33.68 | **33.68** | 0.34 |  |  |
|  | 11 | 4WC2 | 33.47 | 33.83 | 33.44 | **33.58** | 0.22 | 4WControl | |
|  | 12 | 4WC3 | 27.98 | 27.48 | 27.68 | **27.71** | 0.25 | **31.66** | **3.42** |
| Actin (Reference gene) | 1 | 2WS1 | 17.87 | 18.28 | 18.12 | **18.09** | 0.21 |  |  |
|  | 2 | 2WS2 | 19.02 | 18.81 | 19.15 | **18.99** | 0.17 | 2WStress | |
|  | 3 | 2WS3 | 18.51 | 18.43 | 18.66 | **18.53** | 0.12 | **18.54** | **0.45** |
|  | 4 | 2WC1 | 18.70 | 18.44 | 18.37 | **18.50** | 0.17 |  |  |
|  | 5 | 2WC2 | 18.63 | 18.35 | 18.74 | **18.57** | 0.20 | 2WControl | |
|  | 6 | 2WC3 | n/a | n/a | 18.92 | **18.92** | n/a | **18.67** | **0.22** |
|  | 7 | 4WS1 | 18.82 | 18.6 | n/a | **18.71** | 0.16 |  |  |
|  | 8 | 4WS2 | 18.15 | 18.4 | 18.43 | **18.33** | 0.15 | 4WStress | |
|  | 9 | 4WS3 | 18.27 | 18.35 | 18.5 | **18.37** | 0.12 | **18.47** | **0.21** |
|  | 10 | 4WC1 | 18.79 | n/a | n/a | **18.79** | n/a |  |  |
|  | 11 | 4WC2 | 18.13 | 18.16 | 18.23 | **18.17** | 0.05 | 4WControl | |
|  | 12 | 4WC3 | 18.19 | 18.24 | 18.35 | **18.26** | 0.08 | **18.41** | **0.33** |
