# Supplementary material for: Genomic and Epigenomic Responses to Chronic Stress Involve miRNA-Mediated Programming
Source: PLoS One. 2012 Jan 24;7(1):e29441. doi: 10.1371/journal.pone.0029441 (PMC3265462; doi:10.1371/journal.pone.0029441)
Supplement: Table S4 — qRT-PCR data of Adipoq expression in prefrontal cortex. (DOC) [file pone.0029441.s010.doc]

**Table S4.** qRT-PCR data of *Prlr* expression in prefrontal cortex.

| **Gene** | **Sample #** | **Sample name** | **C(t)** | | | **Average C(t)** | **St.dev.** | **Average C(t) and st. dev. from biological repeats** | |
| --- | --- | --- | --- | --- | --- | --- | --- | --- | --- |
| Prlr (Gene of interest) | 1 | 2WS1 | 39.31 | 39.51 | 37.84 | **38.89** | 0.91 |  |  |
|  | 2 | 2WS2 | 38.79 | 39.08 | 40.28 | **39.38** | 0.79 | 2WStress | |
|  | 3 | 2WS3 | 34.84 | 33.25 | 33.84 | **33.98** | 0.80 | **37.42** | **2.99** |
|  | 4 | 2WC1 | 36.38 | 36.58 | 37.01 | **36.66** | 0.32 |  |  |
|  | 5 | 2WC2 | 38.47 | 40.08 | 38.99 | **39.18** | 0.82 | 2WControl | |
|  | 6 | 2WC3 | 36.06 | 37.66 | 23.13 | **32.28** | 7.97 | **36.04** | **3.49** |
|  | 7 | 4WS1 | 33.35 | 36.6 | 33.22 | **34.39** | 1.92 |  |  |
|  | 8 | 4WS2 | 34.33 | 34.51 | 33.26 | **34.03** | 0.68 | 4WStress | |
|  | 9 | 4WS3 | 36.56 | 36.11 | 37.45 | **36.71** | 0.68 | **35.04** | **1.45** |
|  | 10 | 4WC1 | 38.44 | 38.64 | 39.25 | **38.78** | 0.42 |  |  |
|  | 11 | 4WC2 | 39.99 | 39.89 | 38.2 | **39.36** | 1.01 | 4WControl | |
|  | 12 | 4WC3 | 40.50 | 40.16 | 40.24 | **40.30** | 0.18 | **39.48** | **0.77** |
| Actin (Reference gene) | 1 | 2WS1 | 17.77 | 17.82 | 18.54 | **18.04** | 0.43 |  |  |
|  | 2 | 2WS2 | 19.22 | 19.26 | 19.02 | **19.17** | 0.13 | 2WStress | |
|  | 3 | 2WS3 | 18.45 | 18.41 | 18.79 | **18.55** | 0.21 | **18.59** | **0.56** |
|  | 4 | 2WC1 | 19.20 | 18.69 | 18.1 | **18.66** | 0.55 |  |  |
|  | 5 | 2WC2 | 19.42 | 19.29 | 19.59 | **19.43** | 0.15 | 2WControl | |
|  | 6 | 2WC3 | 19.02 | 18.76 | 18.76 | **18.85** | 0.15 | **18.98** | **0.40** |
|  | 7 | 4WS1 | 18.73 | 18.66 | 18.38 | **18.59** | 0.19 |  |  |
|  | 8 | 4WS2 | 18.67 | 19.46 | 19.23 | **19.12** | 0.41 | 4WStress | |
|  | 9 | 4WS3 | 19.45 | 19.26 | 18.97 | **19.23** | 0.24 | **18.98** | **0.34** |
|  | 10 | 4WC1 | 19.72 | 19.75 | 18.99 | **19.49** | 0.43 |  |  |
|  | 11 | 4WC2 | 19.21 | 19.5 | 19.6 | **19.44** | 0.20 | 4WControl | |
|  | 12 | 4WC3 | 19.95 | 19.66 | 19.6 | **19.74** | 0.19 | **19.55** | **0.16** |
