# Supplementary material for: Genomic and Epigenomic Responses to Chronic Stress Involve miRNA-Mediated Programming
Source: PLoS One. 2012 Jan 24;7(1):e29441. doi: 10.1371/journal.pone.0029441 (PMC3265462; doi:10.1371/journal.pone.0029441)
Supplement: Table S5 — Functional annotation clustering analysis of target genes. (DOC) [file pone.0029441.s011.doc]

**Table S5.** qRT-PCR data of *Adipoq* expression in hippocampus.

| **Gene** | **Sample #** | **Sample name** | **C(t)** | | | **Average C(t)** | **St.dev.** | **Average C(t) and st. dev. from biological repeats** | |
| --- | --- | --- | --- | --- | --- | --- | --- | --- | --- |
| Adipoq (Gene of interest) | 1 | 2WS1 | 39.96 | 41.35 | 41.43 | **40.91** | 0.83 |  |  |
|  | 2 | 2WS2 | 45.82 | 46.00 | 46.00 | **45.94** | 0.10 | 2WStress | |
|  | 3 | 2WS3 | 40.84 | 39.79 | 45.08 | **41.90** | 2.80 | **42.92** | **2.66** |
|  | 4 | 2WC1 | 46.00 | 46.00 | 45.42 | **45.81** | 0.33 |  |  |
|  | 5 | 2WC2 | 46.00 | 46.00 | 44.63 | **45.54** | 0.79 | 2WControl | |
|  | 6 | 2WC3 | 41.71 | 39.51 | 41.06 | **40.76** | 1.13 | **44.04** | **2.84** |
|  | 7 | 4WS1 | 46.00 | 46.00 | 46.00 | **46.00** | 0.00 |  |  |
|  | 8 | 4WS2 | 46.00 | 46.00 | 46.00 | **46.00** | 0.00 | 4WStress | |
|  | 9 | 4WS3 | 34.87 | 33.44 | 35.33 | **34.55** | 0.99 | **42.18** |  |
|  | 10 | 4WC1 | 41.12 | 43.13 | 43.1 | **42.45** | 1.15 |  |  |
|  | 11 | 4WC2 | 43.93 | 46.00 | 40.52 | **43.48** | 2.77 | 4WControl | |
|  | 12 | 4WC3 | 34.07 | 35.73 | 35.32 | **35.04** | 0.86 | **40.32** | **4.61** |
| Actin (Reference gene) | 1 | 2WS1 | 18.08 | 18.11 | 19.06 | **18.42** | 0.56 |  |  |
|  | 2 | 2WS2 | 18.17 | 18.45 | 19.1 | **18.57** | 0.48 | 2WStress | |
|  | 3 | 2WS3 | 17.76 | 18.22 | 18.71 | **18.23** | 0.48 | **18.41** | **0.17** |
|  | 4 | 2WC1 | 18.15 | 18.36 | 18.22 | **18.24** | 0.11 |  |  |
|  | 5 | 2WC2 | 17.80 | 18.29 | 19.09 | **18.39** | 0.65 | 2WControl | |
|  | 6 | 2WC3 | 17.65 | 17.58 | 18.85 | **18.03** | 0.71 | **18.22** | **0.18** |
|  | 7 | 4WS1 | 19.26 | 19.28 | 19.8 | **19.45** | 0.31 |  |  |
|  | 8 | 4WS2 | 19.31 | 19.54 | 19.8 | **19.55** | 0.25 | 4WStress | |
|  | 9 | 4WS3 | 19.47 | 19.33 | 20.25 | **19.68** | 0.50 | **19.56** | **0.12** |
|  | 10 | 4WC1 | 46.00 | 19.31 | 19.65 | **28.32** | 15.31 |  |  |
|  | 11 | 4WC2 | 18.67 | 19.0 | 19.42 | **19.03** | 0.38 | 4WControl | |
|  | 12 | 4WC3 | 19.91 | 19.57 | 20.77 | **20.08** | 0.62 | **22.48** | **5.09** |
