# Supplementary material for: Genomic and Epigenomic Responses to Chronic Stress Involve miRNA-Mediated Programming
Source: PLoS One. 2012 Jan 24;7(1):e29441. doi: 10.1371/journal.pone.0029441 (PMC3265462; doi:10.1371/journal.pone.0029441)
Supplement: Table S6 — qRT-PCR data of miR-186 expression in cerebellum. (DOC) [file pone.0029441.s012.doc]

**Table S6.** qRT-PCR data of *Adipoq* expression in prefrontal cortex.

| **Gene** | **Sample #** | **Sample name** | **C(t)** | | | **Average C(t)** | **St.dev.** | **Average C(t) and st. dev. from biological repeats** | |
| --- | --- | --- | --- | --- | --- | --- | --- | --- | --- |
| Adipoq (Gene of interest) | 1 | 2WS1 | 39.86 | 41.27 | 41.34 | **40.82** | 0.84 |  |  |
|  | 2 | 2WS2 | 45.73 | 46.0 | 44.88 | **45.54** | 0.58 | 2WStress | |
|  | 3 | 2WS3 | 40.75 | 39.71 | n/a | **40.23** | 0.74 | **42.20** | **2.91** |
|  | 4 | 2WC1 | 46.0 | 46.0 | 45.33 | **45.78** | 0.39 |  |  |
|  | 5 | 2WC2 | 46.0 | 46.0 | 44.58 | **45.53** | 0.82 | 2WControl | |
|  | 6 | 2WC3 | 41.62 | 39.42 | 40.98 | **40.67** | 1.13 | **43.99** | **2.88** |
|  | 7 | 4WS1 | 46.0 | 46.0 | 46.0 | **46.00** | 0.00 |  |  |
|  | 8 | 4WS2 | 46.0 | 46.0 | 46.0 | **46.00** | 0.00 | 4WStress | |
|  | 9 | 4WS3 | 34.87 | 33.44 | 35.33 | **34.55** | 0.99 | **42.18** |  |
|  | 10 | 4WC1 | 41.12 | 43.13 | 43.1 | **42.45** | 1.15 |  |  |
|  | 11 | 4WC2 | 43.93 | 46.0 | 40.52 | **43.48** | 2.77 | 4WControl | |
|  | 12 | 4WC3 | 34.07 | 35.73 | 35.32 | **35.04** | 0.86 | **40.32** | **4.61** |
| Actin (Reference gene) | 1 | 2WS1 | 18.03 | 18.06 | 19.0 | **18.36** | 0.55 |  |  |
|  | 2 | 2WS2 | 18.12 | 18.4 | 19.05 | **18.52** | 0.48 | 2WStress | |
|  | 3 | 2WS3 | 17.7 | 18.16 | 18.64 | **18.17** | 0.47 | **18.35** | **0.18** |
|  | 4 | 2WC1 | 18.1 | 18.3 | 18.17 | **18.19** | 0.10 |  |  |
|  | 5 | 2WC2 | 17.74 | 18.24 | 19.05 | **18.34** | 0.66 | 2WControl | |
|  | 6 | 2WC3 | 17.59 | 17.52 | 18.78 | **17.96** | 0.71 | **18.17** | **0.19** |
|  | 7 | 4WS1 | 19.26 | 19.28 | 19.8 | **19.45** | 0.31 |  |  |
|  | 8 | 4WS2 | 19.31 | 19.54 | 19.8 | **19.55** | 0.25 | 4WStress | |
|  | 9 | 4WS3 | 19.47 | 19.33 | 20.25 | **19.68** | 0.50 | **19.56** | **0.12** |
|  | 10 | 4WC1 | n/a | 19.31 | 19.65 | **19.48** | 0.24 |  |  |
|  | 11 | 4WC2 | 18.67 | 19.0 | 19.42 | **19.03** | 0.38 | 4WControl | |
|  | 12 | 4WC3 | 19.91 | 19.57 | 20.77 | **20.08** | 0.62 | **19.53** | **0.53** |
