# Supplementary material for: Genomic and Epigenomic Responses to Chronic Stress Involve miRNA-Mediated Programming
Source: PLoS One. 2012 Jan 24;7(1):e29441. doi: 10.1371/journal.pone.0029441 (PMC3265462; doi:10.1371/journal.pone.0029441)
Supplement: Table S7 — qRT-PCR data of miR-709 expression in cerebellum. (DOC) [file pone.0029441.s013.doc]

**Table S7.** qRT-PCR data of miR-186 expression in cerebellum.

| **Gene** | **Sample #** | **Sample name** | **C(t)** | | | **Average C(t)** | **St.dev.** | **Average C(t) and st. dev. from biological repeats** | |
| --- | --- | --- | --- | --- | --- | --- | --- | --- | --- |
| miR-186 (Gene of interest) | 1 | 2WS1 | 35.99 | 35.43 | 35.3 | **35.57** | 0.37 |  |  |
|  | 2 | 2WS2 | 34.54 | 34.4 | 34.15 | **34.36** | 0.20 | 2WStress | |
|  | 3 | 2WS3 | 34.47 | 34.56 | 34.1 | **34.38** | 0.24 | **34.77** | **0.69** |
|  | 4 | 2WC1 | 35.1 | 35.2 | 35.34 | **35.21** | 0.12 |  |  |
|  | 5 | 2WC2 | 34.44 | 34.55 | 34.33 | **34.44** | 0.11 | 2WControl | |
|  | 6 | 2WC3 | 35.45 | 36.27 | 35.7 | **35.81** | 0.42 | **35.15** | **0.69** |
|  | 7 | 4WS1 | 33.28 | 33.5 | 33.97 | **33.58** | 0.35 |  |  |
|  | 8 | 4WS2 | 34.46 | 33.88 | 34.3 | **34.21** | 0.30 | 4WStress | |
|  | 9 | 4WS3 | 33.97 | 34.18 | 33.92 | **34.02** | 0.14 | **33.94** | **0.32** |
|  | 10 | 4WC1 | 35.69 | 35.16 | 35.28 | **35.38** | 0.28 |  |  |
|  | 11 | 4WC2 | 34.91 | 34.94 | 34.45 | **34.77** | 0.27 | 4WControl | |
|  | 12 | 4WC3 | 36.38 | 36.54 | 36.47 | **36.46** | 0.08 | **35.54** | **0.86** |
| Rnu-6 (Reference gene) | 1 | 2WS1 | 23.83 | 23.63 | 23.52 | **23.66** | 0.16 |  |  |
|  | 2 | 2WS2 | 23.37 | 23.04 | 23.2 | **23.20** | 0.17 | 2WStress | |
|  | 3 | 2WS3 | 23.15 | 23.22 | 23.07 | **23.15** | 0.08 | **23.34** | **0.28** |
|  | 4 | 2WC1 | 24.62 | 24.64 | 24.52 | **24.59** | 0.06 |  |  |
|  | 5 | 2WC2 | 23.54 | 24.07 | 23.41 | **23.67** | 0.35 | 2WControl | |
|  | 6 | 2WC3 | 23.51 | 23.55 | 23.4 | **23.49** | 0.08 | **23.92** | **0.59** |
|  | 7 | 4WS1 | 22.88 | 23.21 | 22.84 | **22.98** | 0.20 |  |  |
|  | 8 | 4WS2 | 23.11 | 23.11 | 23.24 | **23.15** | 0.08 | 4WStress | |
|  | 9 | 4WS3 | 23.02 | 23.0 | 23.12 | **23.05** | 0.06 | **23.06** | **0.09** |
|  | 10 | 4WC1 | 24.68 | 24.76 | 24.76 | **24.73** | 0.05 |  |  |
|  | 11 | 4WC2 | 23.23 | 23.42 | 23.39 | **23.35** | 0.10 | 4WControl | |
|  | 12 | 4WC3 | 24.0 | 24.0 | 24.03 | **24.01** | 0.02 | **24.03** | **0.69** |
