# Supplementary material for: Genomic and Epigenomic Responses to Chronic Stress Involve miRNA-Mediated Programming
Source: PLoS One. 2012 Jan 24;7(1):e29441. doi: 10.1371/journal.pone.0029441 (PMC3265462; doi:10.1371/journal.pone.0029441)
Supplement: Table S8 — qRT-PCR data of miR-186 expression in hippocampus. (DOC) [file pone.0029441.s014.doc]

**Table S8.** qRT-PCR data of miR-709 expression in cerebellum.

| **Gene** | **Sample #** | **Sample name** | **C(t)** | | | **Average C(t)** | **St.dev.** | **Average C(t) and st. dev. from biological repeats** | |
| --- | --- | --- | --- | --- | --- | --- | --- | --- | --- |
| miR-709 (Gene of interest) | 1 | 2WS1 | 26.87 | 26.51 | 26.66 | **26.68** | 0.18 |  |  |
|  | 2 | 2WS2 | 26.76 | 26.56 | 26.5 | **26.61** | 0.14 | 2WStress | |
|  | 3 | 2WS3 | 26.88 | 28.99 | 26.45 | **27.44** | 1.36 | **26.91** | **0.46** |
|  | 4 | 2WC1 | 26.87 | 26.48 | 26.56 | **26.64** | 0.21 |  |  |
|  | 5 | 2WC2 | 27.27 | 26.75 | 26.68 | **26.90** | 0.32 | 2WControl | |
|  | 6 | 2WC3 | 26.3 | 26.95 | 25.85 | **26.37** | 0.55 | **26.63** | **0.27** |
|  | 7 | 4WS1 | 28.45 | 26.67 | 26.68 | **27.27** | 1.02 |  |  |
|  | 8 | 4WS2 | 27.67 | 28.14 | 28.4 | **28.07** | 0.37 | 4WStress | |
|  | 9 | 4WS3 | 27.05 | 27.13 | 27.18 | **27.12** | 0.07 | **27.49** | **0.51** |
|  | 10 | 4WC1 | 26.97 | n/a | 26.71 | **26.84** | 0.18 |  |  |
|  | 11 | 4WC2 | 26.76 | 26.12 | 26.18 | **26.35** | 0.35 | 4WControl | |
|  | 12 | 4WC3 | 27.29 | 27.05 | 27.32 | **27.22** | 0.15 | **26.80** | **0.43** |
| Rnu-6 (Reference gene) | 1 | 2WS1 | 23.63 | 23.55 | 23.45 | **23.54** | 0.09 |  |  |
|  | 2 | 2WS2 | 23.15 | 23.02 | 22.98 | **23.05** | 0.09 | 2WStress | |
|  | 3 | 2WS3 | 22.85 | 22.74 | 22.66 | **22.75** | 0.10 | **23.11** | **0.40** |
|  | 4 | 2WC1 | 24.47 | 24.47 | 24.27 | **24.40** | 0.12 |  |  |
|  | 5 | 2WC2 | 23.36 | 23.19 | 23.11 | **23.22** | 0.13 | 2WControl | |
|  | 6 | 2WC3 | 23.4 | 23.21 | 23.2 | **23.27** | 0.11 | **23.63** | **0.67** |
|  | 7 | 4WS1 | 22.99 | 23.03 | 22.87 | **22.96** | 0.08 |  |  |
|  | 8 | 4WS2 | 23.15 | 23.03 | 23.01 | **23.06** | 0.08 | 4WStress | |
|  | 9 | 4WS3 | 22.81 | 22.79 | 22.92 | **22.84** | 0.07 | **22.96** | **0.11** |
|  | 10 | 4WC1 | 24.61 | 24.5 | 24.51 | **24.54** | 0.06 |  |  |
|  | 11 | 4WC2 | 23.16 | 23.2 | 23.14 | **23.17** | 0.03 | 4WControl | |
|  | 12 | 4WC3 | 23.93 | 23.84 | 23.92 | **23.90** | 0.05 | **23.87** | **0.69** |
