# Supplementary material for: Genomic and Epigenomic Responses to Chronic Stress Involve miRNA-Mediated Programming
Source: PLoS One. 2012 Jan 24;7(1):e29441. doi: 10.1371/journal.pone.0029441 (PMC3265462; doi:10.1371/journal.pone.0029441)
Supplement: Table S9 — qRT-PCR data of miR-709 expression in hippocampus. (DOC) [file pone.0029441.s015.doc]

**Table S9.** qRT-PCR data of miR-186 expression in hippocampus.

| **Gene** | **Sample #** | **Sample name** | **C(t)** | | | **Average C(t)** | **St.dev.** | **Average C(t) and st. dev. from biological repeats** | |
| --- | --- | --- | --- | --- | --- | --- | --- | --- | --- |
| miR-186 (Gene of interest) | 1 | 2WS1 | 30.01 | 29.73 | 30.15 | **29.96** | 0.21 |  |  |
|  | 2 | 2WS2 | 30.34 | 30.57 | 30.45 | **30.45** | 0.12 | 2WStress | |
|  | 3 | 2WS3 | 30.45 | 30.58 | 30.57 | **30.53** | 0.07 | **30.32** | **0.31** |
|  | 4 | 2WC1 | 30.67 | 30.47 | 30.35 | **30.50** | 0.16 |  |  |
|  | 5 | 2WC2 | 30.48 | 30.43 | 30.26 | **30.39** | 0.12 | 2WControl | |
|  | 6 | 2WC3 | 30.14 | 29.98 | 29.9 | **30.01** | 0.12 | **30.30** | **0.26** |
|  | 7 | 4WS1 | 30.02 | 29.8 | 29.96 | **29.93** | 0.11 |  |  |
|  | 8 | 4WS2 | 30.36 | 29.98 | 30.12 | **30.15** | 0.19 | 4WStress | |
|  | 9 | 4WS3 | 30.2 | 30.11 | 30.05 | **30.12** | 0.08 | **30.07** | **0.12** |
|  | 10 | 4WC1 | 29.34 | 29.16 | 29.0 | **29.17** | 0.17 |  |  |
|  | 11 | 4WC2 | 30.25 | 30.23 | 30.06 | **30.18** | 0.10 | 4WControl | |
|  | 12 | 4WC3 | 30.28 | 30.16 | 30.37 | **30.27** | 0.11 | **29.87** | **0.61** |
| Rnu-6 (Reference gene) | 1 | 2WS1 | 20.19 | 20.29 | 20.26 | **20.25** | 0.05 |  |  |
|  | 2 | 2WS2 | 21.45 | 21.45 | 21.41 | **21.44** | 0.02 | 2WStress | |
|  | 3 | 2WS3 | 22.04 | 21.95 | 21.8 | **21.93** | 0.12 | **21.20** | **0.87** |
|  | 4 | 2WC1 | 19.25 | 19.29 | 19.35 | **19.30** | 0.05 |  |  |
|  | 5 | 2WC2 | 21.24 | 21.22 | 21.3 | **21.25** | 0.04 | 2WControl | |
|  | 6 | 2WC3 | 21.57 | 21.66 | 21.52 | **21.58** | 0.07 | **20.71** | **1.24** |
|  | 7 | 4WS1 | 21.05 | 21.15 | 21.02 | **21.07** | 0.07 |  |  |
|  | 8 | 4WS2 | 21.45 | 21.16 | 21.21 | **21.27** | 0.16 | 4WStress | |
|  | 9 | 4WS3 | 21.46 | 21.48 | 21.45 | **21.46** | 0.02 | **21.27** | **0.20** |
|  | 10 | 4WC1 | 18.93 | 18.92 | 18.74 | **18.86** | 0.11 |  |  |
|  | 11 | 4WC2 | 21.3 | 21.42 | 21.56 | **21.43** | 0.13 | 4WControl | |
|  | 12 | 4WC3 | 21.24 | 21.28 | 21.14 | **21.22** | 0.07 | **20.50** | **1.42** |
