# Supplementary material for: Genomic and Epigenomic Responses to Chronic Stress Involve miRNA-Mediated Programming
Source: PLoS One. 2012 Jan 24;7(1):e29441. doi: 10.1371/journal.pone.0029441 (PMC3265462; doi:10.1371/journal.pone.0029441)
Supplement: Table S10 — qRT-PCR data of miR-186 expression in prefrontal cortex. (DOC) [file pone.0029441.s016.doc]

**Table S10.** qRT-PCR data of miR-709 expression in hippocampus.

| **Gene** | **Sample #** | **Sample name** | **C(t)** | | | **Average C(t)** | **St.dev.** | **Average C(t) and st. dev. from biological repeats** | |
| --- | --- | --- | --- | --- | --- | --- | --- | --- | --- |
| miR-709 (Gene of interest) | 1 | 2WS1 | 27.73 | 28.15 | 28.06 | **27.98** | 0.22 |  |  |
|  | 2 | 2WS2 | 28.42 | 27.24 | 26.86 | **27.51** | 0.81 | 2WStress | |
|  | 3 | 2WS3 | 26.29 | 25.81 | 26.12 | **26.07** | 0.24 | **27.19** | **0.99** |
|  | 4 | 2WC1 | 27.65 | 27.49 | 27.93 | **27.69** | 0.22 |  |  |
|  | 5 | 2WC2 | 27.49 | 27.36 | 27.17 | **27.34** | 0.16 | 2WControl | |
|  | 6 | 2WC3 | 27.92 | 28.4 | 27.79 | **28.04** | 0.32 | **27.69** | **0.35** |
|  | 7 | 4WS1 | 27.31 | 27.1 | 27.28 | **27.23** | 0.11 |  |  |
|  | 8 | 4WS2 | 27.61 | 27.24 | 27.38 | **27.41** | 0.19 | 4WStress | |
|  | 9 | 4WS3 | 26.37 | 26.1 | 26.21 | **26.23** | 0.14 | **26.96** | **0.64** |
|  | 10 | 4WC1 | 26.27 | 26.28 | 25.91 | **26.15** | 0.21 |  |  |
|  | 11 | 4WC2 | 27.0 | 27.16 | 27.07 | **27.08** | 0.08 | 4WControl | |
|  | 12 | 4WC3 | 27.23 | 27.1 | 27.22 | **27.18** | 0.07 | **26.80** | **0.57** |
| Rnu-6 (Reference gene) | 1 | 2WS1 | 20.01 | 20.07 | 20.01 | **20.03** | 0.03 |  |  |
|  | 2 | 2WS2 | 21.26 | 21.1 | 21.26 | **21.21** | 0.09 | 2WStress | |
|  | 3 | 2WS3 | 21.74 | 21.62 | 21.49 | **21.62** | 0.13 | **20.95** | **0.82** |
|  | 4 | 2WC1 | 19.47 | 19.11 | 19.0 | **19.19** | 0.25 |  |  |
|  | 5 | 2WC2 | 21.01 | 20.76 | 20.75 | **20.84** | 0.15 | 2WControl | |
|  | 6 | 2WC3 | 21.29 | 21.24 | 21.3 | **21.28** | 0.03 | **20.44** | **1.10** |
|  | 7 | 4WS1 | 21.16 | 21.07 | 20.99 | **21.07** | 0.09 |  |  |
|  | 8 | 4WS2 | 21.17 | 21.08 | 21.1 | **21.12** | 0.05 | 4WStress | |
|  | 9 | 4WS3 | 21.33 | 21.35 | 21.4 | **21.36** | 0.04 | **21.18** | **0.15** |
|  | 10 | 4WC1 | 18.85 | 18.8 | 18.62 | **18.76** | 0.12 |  |  |
|  | 11 | 4WC2 | 21.33 | 21.2 | 21.27 | **21.27** | 0.07 | 4WControl | |
|  | 12 | 4WC3 | 21.3 | 21.05 | 20.94 | **21.10** | 0.18 | **20.37** | **1.40** |
