# Supplementary material for: Genomic and Epigenomic Responses to Chronic Stress Involve miRNA-Mediated Programming
Source: PLoS One. 2012 Jan 24;7(1):e29441. doi: 10.1371/journal.pone.0029441 (PMC3265462; doi:10.1371/journal.pone.0029441)
Supplement: Table S11 — qRT-PCR data of miR-709 expression in prefrontal cortex. (DOC) [file pone.0029441.s017.doc]

**Table S11.** qRT-PCR data of miR-186 expression in prefrontal cortex.

| **Gene** | **Sample #** | **Sample name** | **C(t)** | | | **Average C(t)** | **St.dev.** | **Average C(t) and st. dev. from biological repeats** | |
| --- | --- | --- | --- | --- | --- | --- | --- | --- | --- |
| miR-186 (Gene of interest) | 1 | 2WS1 | 29.84 | 30.14 | 30.08 | **30.02** | 0.16 |  |  |
|  | 2 | 2WS2 | 30.02 | 29.85 | 29.85 | **29.91** | 0.10 | 2WStress | |
|  | 3 | 2WS3 | 30.52 | 30.67 | 30.4 | **30.53** | 0.14 | **30.15** | **0.33** |
|  | 4 | 2WC1 | 30.79 | 30.98 | 30.86 | **30.88** | 0.10 |  |  |
|  | 5 | 2WC2 | 30.83 | 30.8 | 30.68 | **30.77** | 0.08 | 2WControl | |
|  | 6 | 2WC3 | 31.1 | 31.0 | 30.83 | **30.98** | 0.14 | **30.87** | **0.10** |
|  | 7 | 4WS1 | 30.62 | 30.17 | 30.39 | **30.39** | 0.23 |  |  |
|  | 8 | 4WS2 | 30.69 | 30.65 | 31.0 | **30.78** | 0.19 | 4WStress | |
|  | 9 | 4WS3 | 30.73 | 30.87 | 30.75 | **30.78** | 0.08 | **30.65** | **0.22** |
|  | 10 | 4WC1 | 31.47 | 31.12 | 31.23 | **31.27** | 0.18 |  |  |
|  | 11 | 4WC2 | 31.98 | 31.47 | 31.46 | **31.64** | 0.30 | 4WControl | |
|  | 12 | 4WC3 | 31.71 | 31.51 | 31.48 | **31.57** | 0.13 | **31.49** | **0.19** |
| Rnu-6 (Reference gene) | 1 | 2WS1 | 21.02 | 21.16 | 21.04 | **21.07** | 0.08 |  |  |
|  | 2 | 2WS2 | 19.99 | 19.86 | 19.84 | **19.90** | 0.08 | 2WStress | |
|  | 3 | 2WS3 | 21.08 | 20.92 | 21.02 | **21.01** | 0.08 | **20.66** | **0.66** |
|  | 4 | 2WC1 | 20.83 | 20.72 | 20.82 | **20.79** | 0.06 |  |  |
|  | 5 | 2WC2 | 20.63 | 20.49 | 20.54 | **20.55** | 0.07 | 2WControl | |
|  | 6 | 2WC3 | 21.23 | 21.09 | 21.05 | **21.12** | 0.09 | **20.82** | **0.29** |
|  | 7 | 4WS1 | 21.33 | 21.29 | 21.23 | **21.28** | 0.05 |  |  |
|  | 8 | 4WS2 | 21.07 | 20.9 | 20.91 | **20.96** | 0.10 | 4WStress | |
|  | 9 | 4WS3 | 21.12 | 20.97 | 21.0 | **21.03** | 0.08 | **21.09** | **0.17** |
|  | 10 | 4WC1 | 19.99 | 19.94 | 19.82 | **19.92** | 0.09 |  |  |
|  | 11 | 4WC2 | 21.09 | 21.18 | 21.2 | **21.16** | 0.06 | 4WControl | |
|  | 12 | 4WC3 | 21.38 | 21.28 | 21.33 | **21.33** | 0.05 | **20.80** | **0.77** |
