# Supplementary material for: Genomic and Epigenomic Responses to Chronic Stress Involve miRNA-Mediated Programming
Source: PLoS One. 2012 Jan 24;7(1):e29441. doi: 10.1371/journal.pone.0029441 (PMC3265462; doi:10.1371/journal.pone.0029441)
Supplement: Table S12 — Primers and PCR conditions for sq-RT-PCR, qRT-PCR, and cloning. (DOCX) [file pone.0029441.s018.docx]

**Table S12.** qRT-PCR data of miR-709 expression in prefrontal cortex.

| **Gene** | **Sample #** | **Sample name** | **C(t)** | | | **Average C(t)** | **St.dev.** | **Average C(t) and st. dev. from biological repeats** | |
| --- | --- | --- | --- | --- | --- | --- | --- | --- | --- |
| miR-709 (Gene of interest) | 1 | 2WS1 | 27.44 | 26.37 | 26.08 | **26.63** | 0.72 |  |  |
|  | 2 | 2WS2 | 27.2 | 26.07 | 26.68 | **26.65** | 0.57 | 2WStress | |
|  | 3 | 2WS3 | 27.26 | 27.08 | 27.19 | **27.18** | 0.09 | **26.82** | **0.31** |
|  | 4 | 2WC1 | 26.71 | 26.34 | 26.43 | **26.49** | 0.19 |  |  |
|  | 5 | 2WC2 | 27.63 | 27.25 | 26.98 | **27.29** | 0.33 | 2WControl | |
|  | 6 | 2WC3 | 28.01 | 27.07 | 27.27 | **27.45** | 0.50 | **27.08** | **0.51** |
|  | 7 | 4WS1 | 26.12 | 25.89 | 26.04 | **26.02** | 0.12 |  |  |
|  | 8 | 4WS2 | 26.19 | 26.21 | 26.17 | **26.19** | 0.02 | 4WStress | |
|  | 9 | 4WS3 | 27.05 | 26.37 | 26.54 | **26.65** | 0.35 | **26.29** | **0.33** |
|  | 10 | 4WC1 | 26.23 | 26.81 | 26.38 | **26.47** | 0.30 |  |  |
|  | 11 | 4WC2 | 28.08 | 26.74 | 26.47 | **27.10** | 0.86 | 4WControl | |
|  | 12 | 4WC3 | 27.07 | 26.4 | 28.03 | **27.17** | 0.82 | **26.91** | **0.38** |
| Rnu-6 (Reference gene) | 1 | 2WS1 | 20.12 | 20.27 | 20.14 | **20.18** | 0.08 |  |  |
|  | 2 | 2WS2 | 21.34 | 21.18 | 21.26 | **21.26** | 0.08 | 2WStress | |
|  | 3 | 2WS3 | 21.37 | 21.47 | 21.35 | **21.40** | 0.06 | **20.94** | **0.67** |
|  | 4 | 2WC1 | 21.13 | 21.07 | 21.22 | **21.14** | 0.08 |  |  |
|  | 5 | 2WC2 | 21.2 | 21.24 | 21.01 | **21.15** | 0.12 | 2WControl | |
|  | 6 | 2WC3 | 21.53 | 21.57 | 21.5 | **21.53** | 0.04 | **21.27** | **0.22** |
|  | 7 | 4WS1 | 21.76 | 21.68 | 21.69 | **21.71** | 0.04 |  |  |
|  | 8 | 4WS2 | 21.33 | 21.92 | 21.44 | **21.56** | 0.31 | 4WStress | |
|  | 9 | 4WS3 | 21.25 | 21.39 | 21.31 | **21.32** | 0.07 | **21.53** | **0.20** |
|  | 10 | 4WC1 | 20.27 | 20.36 | 20.27 | **20.30** | 0.05 |  |  |
|  | 11 | 4WC2 | 21.56 | 21.72 | 21.29 | **21.52** | 0.22 | 4WControl | |
|  | 12 | 4WC3 | 21.59 | 21.76 | 21.61 | **21.65** | 0.09 | **21.16** | **0.75** |
